# Does poor methodological quality of prediction modeling studies translate to poor model performance? An illustration in traumatic brain injury

**DOI:** 10.1186/s41512-022-00122-0

**Published:** 2022-05-05

**Authors:** Isabel R. A. Retel Helmrich, Ana Mikolić, David M. Kent, Hester F. Lingsma, Laure Wynants, Ewout W. Steyerberg, David van Klaveren

**Affiliations:** 1 Department of Public Health, Center for Medical Decision Making, Erasmus MC-University Medical Center, Rotterdam, the Netherlands; 2 Predictive Analytics and Comparative Effectiveness Center, Institute for Clinical Research and Health Policy Studies/Tufts Medical Center, Boston, USA; 3 Department of Epidemiology, School for Public Health and Primary Care, Faculty of Health, Medicine and Life Sciences, Maastricht University, Maastricht, The Netherlands; 4 Department of Biomedical Data Sciences, Leiden University Medical Center, Leiden, The Netherlands

**Keywords:** Prognostic model studies, Traumatic brain injury, PROBAST

## Abstract

**Background:**

Prediction modeling studies often have methodological limitations, which may compromise model performance in new patients and settings. We aimed to examine the relation between methodological quality of model development studies and their performance at external validation.

**Methods:**

We systematically searched for externally validated multivariable prediction models that predict functional outcome following moderate or severe traumatic brain injury. Risk of bias and applicability of development studies was assessed with the Prediction model Risk Of Bias Assessment Tool (PROBAST). Each model was rated for its presentation with sufficient detail to be used in practice. Model performance was described in terms of discrimination (AUC), and calibration. Delta AUC (dAUC) was calculated to quantify the percentage change in discrimination between development and validation for all models. Generalized estimation equations (GEE) were used to examine the relation between methodological quality and dAUC while controlling for clustering.

**Results:**

We included 54 publications, presenting ten development studies of 18 prediction models, and 52 external validation studies, including 245 unique validations. Two development studies (four models) were found to have low risk of bias (RoB). The other eight publications (14 models) showed high or unclear RoB. The median dAUC was positive in low RoB models (dAUC 8%, [IQR − 4% to 21%]) and negative in high RoB models (dAUC − 18%, [IQR − 43% to 2%]). The GEE showed a larger average negative change in discrimination for high RoB models (− 32% (95% CI: − 48 to − 15) and unclear RoB models (− 13% (95% CI: − 16 to − 10)) compared to that seen in low RoB models.

**Conclusion:**

Lower methodological quality at model development associates with poorer model performance at external validation. Our findings emphasize the importance of adherence to methodological principles and reporting guidelines in prediction modeling studies.

**Supplementary Information:**

The online version contains supplementary material available at 10.1186/s41512-022-00122-0.

## Introduction

Prediction models estimate an individual’s risk of a certain outcome based on a combination of (clinical) characteristics. Despite numerous efforts to provide guidelines and recommendations for the reporting and analyses of prediction modeling studies [1, 2], these studies often suffer from methodological limitations. Prior reviews have judged the methodological quality of prediction modeling studies generally as poor [3–5], due to the small sample size of the derivation cohort, and a lack of internal and external validation. Furthermore, prediction modeling studies often suffer from incomplete reporting, which could indicate that specific methodological aspects were not considered.

Prognostic models that predict functional outcome after moderate and severe traumatic brain injury (TBI) are abundant in the literature; 67 prognostic models for moderate and severe TBI have been developed, of which 31 were externally validated over the past decades [6]. The ability to accurately predict patient outcome after traumatic brain injury (TBI) has an important role in clinical practice and research. Outcome prognostication may support clinicians in providing reliable information to patients and relatives, and guide clinical management and study design.

Satisfactory methodological quality of prediction modeling studies is considered a prerequisite before implementation of the model in clinical practice should be advocated. Usability of a prediction model, which could be determined by whether sufficient information is provided about the model to enable use in practice, is expected to stimulate its implementation. The reporting of the full model equation enables validation, whereas the development of an online calculator might facilitate use in clinical practice. Assessing the quality of included studies and model usability are therefore important steps in systematic reviews of prediction models.

Recently, the Prediction model Risk Of Bias Assessment Tool (PROBAST) tool has become available to assess the risk of bias and concerns regarding applicability of studies that develop and/or validate a multivariable prediction model in systematic reviews [7]. Risk of bias indicates that shortcomings in the study design, conduct, or analysis may lead to systematically distorted estimates of model predictive performance. Methodological quality of prediction modeling studies might therefore be related to model performance, with lower methodological quality resulting in poor performance, especially in new patients, and settings.

The aim of our study was to empirically examine the relation between the methodological quality of a model development study and model performance at external validation.

## Methods

### Systematic search

We used data from a recent systematic review of multivariable prediction models based on admission characteristics (first 24 h after injury), for patients after moderate and severe TBI (Glasgow Coma Scale ≤ 12) that were published between 2006 and 2018 [6] (Supplementary Table 1 and 2). The protocol of this systematic review has been registered on PROSPERO (registration number 2016: CRD42016052100). Studies were eligible for inclusion if they reported on the development, validation, or extension of multivariable prognostic models for functional outcome in patients aged ≥ 14 years with moderate and severe TBI. There were no limitations concerning outcome measurement, provided that functional outcome was measured between 14 days and 24 months after injury.

We updated the systematic search for 2019–2021 (December 2018–June 2021). One investigator (IRH) independently screened records for possibly relevant studies based on title and abstract. Subsequently, full texts of potentially relevant articles were assessed for eligibility. In case of doubt, a second investigator (AM) was consulted.

### Study selection

We selected externally validated prediction models for moderate and severe TBI (Supplement Table 1) as previously identified by Dijkland et al. (2019) or identified through the updated search. To be included, the model development study had to report model performance in terms of discriminative ability. The external validation could be described in the same publication that described model development, or in a separate publication.

**Table 1:** Methodological quality of model development studies for outcome following moderate and severe traumatic brain injury in terms of applicability and risk of bias assessed with the original PROBAST and models’ usability in research and clinical practice

Study	Models	Applicability				Risk of bias					Usability	
		Participant selection	Predictors	Outcome	Overall applicability	Participant selection	Predictors	Outcome	Analysis	Overall RoB	Research	Practice
Knaus	APACHE II	H	L	L	H	L	L	L	H	H	y	y
Le Gall	SAPS II	H	L	L	H	L	L	L	H	H	n	y
Lemeshow	MPM II models	H	L	L	H	L	L	L	H	H	n	n
Signorini	Signorini	L	L	L	L	L	L	L	H	H	n	y
Hukkelhoven	Hukkelhoven model	L	L	L	L	L	L	L	L	L	y	y
Maas	Rotterdam CT score	L	L	L	L	L	L	L	U	U	n	y
Perel	CRASH models	L	L	L	L	L	L	L	U	U	n	y
Steyerberg	IMPACT models	L	L	L	L	L	L	L	L	L	y	y
Jacobs	Nijmegen models	L	L	L	L	L	L	L	H	H	y	y
Yuan	Yuan models	L	L	L	L	L	U	L	H	H	n	y

Risk of Bias: Low = L; High = H; Unclear = U

Usability: No = n; Yes = y

All models within the same publication were judged the same on applicability, risk of bias and usability and therefore results are reported per publication

### Data extraction

One investigator (IRH) extracted data from the included studies. A check for all included studies was performed by a second investigator (AM). For the development studies, the data extraction form was based on the Critical Appraisal and Data Extraction for Systematic Reviews of Prediction Modeling Studies (CHARMS) checklist [8], and included the source of data, participants, outcome, sample size, predictors, missing data, model development, performance measures, and presentation. For the validation studies, data was extracted on the study design, setting, inclusion criteria, sample size, and model performance. To ensure consistency of the data extraction, the form was tested on two studies by both investigators.

If one publication reported on multiple prediction models, data extraction was performed separately for each model. Prediction models were classified as separate if they included a different set of predictors (e.g., IMPACT core, and IMPACT extended [9]). Models with identical set of predictors, but for different outcomes (e.g., mortality and unfavorable outcome) were not classified as separate models.

### Risk of bias and applicability

Risk of bias and applicability of included development studies were assessed with the Prediction model Risk Of Bias Assessment Tool (PROBAST) [7]. Judgments on high, low, or unclear risk of bias for the model development studies were made for five key domains (participant selection, predictors, outcome, sample size and participant flow, and analysis) using 20 signaling questions (Supplementary Table 3). We also used a short form based on the PROBAST including 8/20 signaling questions, which was recently proposed and validated, and showed high sensitivity (98%) and perfect specificity to identify high risk of bias (RoB) [10].

To determine if there was a reasonable number of outcome events in a logistic regression (PROBAST item 4.1), the lowest number of events in the smallest group of two outcome frequencies (patients with the outcome versus without the outcome) was divided by the total degrees of freedom used during the whole modeling process. The total degrees of freedom was based on the number of variables (continuous variables) or categories (categorical variables) in the model; henceforth referred to as Events Per Parameter (EPP). All candidate predictors were considered as part of the modeling process, including those not selected for the multivariable model based on univariable regression analysis or selection procedures. We assumed a reasonable number of outcome events when EPP ≥ 10.

Concerns regarding the applicability of an included study to the review question can arise when the population, predictors, or outcomes of the included study differ from those specified in the review question [7]. Applicability was judged based on three key domains (participant selection, predictors, and outcome).

Two reviewers (IRH and AM) independently completed the PROBAST checklist (Supplementary Table 3). A third independent reviewer (LW) scored two of the model development studies (17%). Discrepancies between reviewers were resolved through discussion or by consultation with a senior member (DvK) of the review team. The RoB, applicability, and usability were reported per study, in which we presented one assessment for models described in the same publication, but with a different set of predictors (e.g., IMPACT core, and IMPACT extended) and models with identical set of predictors, but for different outcomes (e.g., mortality and unfavorable outcome). An overall judgment about risk of bias and applicability of the prediction model study was reached based on a summative rating across all domains according to the PROBAST criteria (low, high, or unclear).

### Usability

A model’s usability in research and clinical practice was rated for its presentation with sufficient detail to be used in the intended context and target population. The model was deemed usable in research if the full model equation or sufficient information to extract the baseline risk (intercept) and individual predictor effects was reported, and usable in clinical practice if an alternative presentation of the model was included (e.g., a nomogram, score chart, or web calculator).

### Relatedness

For validation studies, we assessed the similarity between the derivation population and the validation population for each study, which we refer to as “relatedness.” To judge relatedness, we created a rubric, aiming to capture various levels or relatedness by dividing the validation studies into three categories: “related,” “moderately related,” and “distantly related” (6) (Supplementary Table 4). The rubric contained three domains: (I) setting (Intensive Care Unit, Emergency Department, Ward; Country; Not specified), (II) inclusion criteria, and (III) outcome assessment and timing. Studies that did not meet the domain about setting were judged “moderately related,” whereas studies that did not meet the domains about inclusion criteria and/or outcome assessment and timing were judged “distantly related.”

### Model performance

Model performance was summarized in terms of discrimination and calibration. In prior studies, discrimination was assessed in terms of the c statistic or area under the operating receiver curve (AUC), which ranges between 0.50 (no discrimination) and 1.0 (perfect discrimination). In prior studies, calibration was typically assessed with the calibration intercept *a*, which indicates whether predictions are systematically too low or too high, and should ideally be 0. Prior studies also reported the calibration slope *b* which indicates whether the overall prognostic effect of the linear predictor of the developed model is over- or underestimated, and should ideally be 1.

### Relation between methodological quality and model performance

To quantify the relation between methodological quality at development and model performance at external validation, we first calculated the change in discriminative performance between the derivation cohort and the validation cohort. The percent change in discrimination was calculated as follows:

$$ \%\mathrm{change}\ \mathrm{in}\ \mathrm{discrimination}=\frac{\left(\mathrm{validation}\ \mathrm{AUC}-0.5\right)-\left(\mathrm{derivation}\ \mathrm{AUC}-0.5\right)}{\left(\mathrm{derivation}\ \mathrm{AUC}-0.5\right)}\ \mathrm{x}\ 100 $$

For instance, when the AUC decreases from 0.70 in derivation to 0.60 in validation, this drop of 0.10 points represents a 50% loss in discriminative power (since 0.50 represents the lowest possible value). We calculated the median and interquartile range (IQR) of the change in discrimination for low, high and unclear RoB models.

We used generalized estimated equations (GEE) to estimate the effect of the RoB classification (Low; High; Unclear RoB based on the original PROBAST) on the observed change in discrimination, taking into account the correlation between validations of the same model and similarity in study design between the development and validation study (Similar; Cohort to trial; Trial to cohort).

### Evidence synthesis

A synthesis was provided for the included development and external validation studies. Extracted data, RoB, applicability, and usability were presented in summary tables and where appropriate in graphical representations. Figures were constructed with R software version 3.6 (R Foundation for Statistical Computing, Vienna, Austria).

## Results

### Study selection

**Fig. 1:**
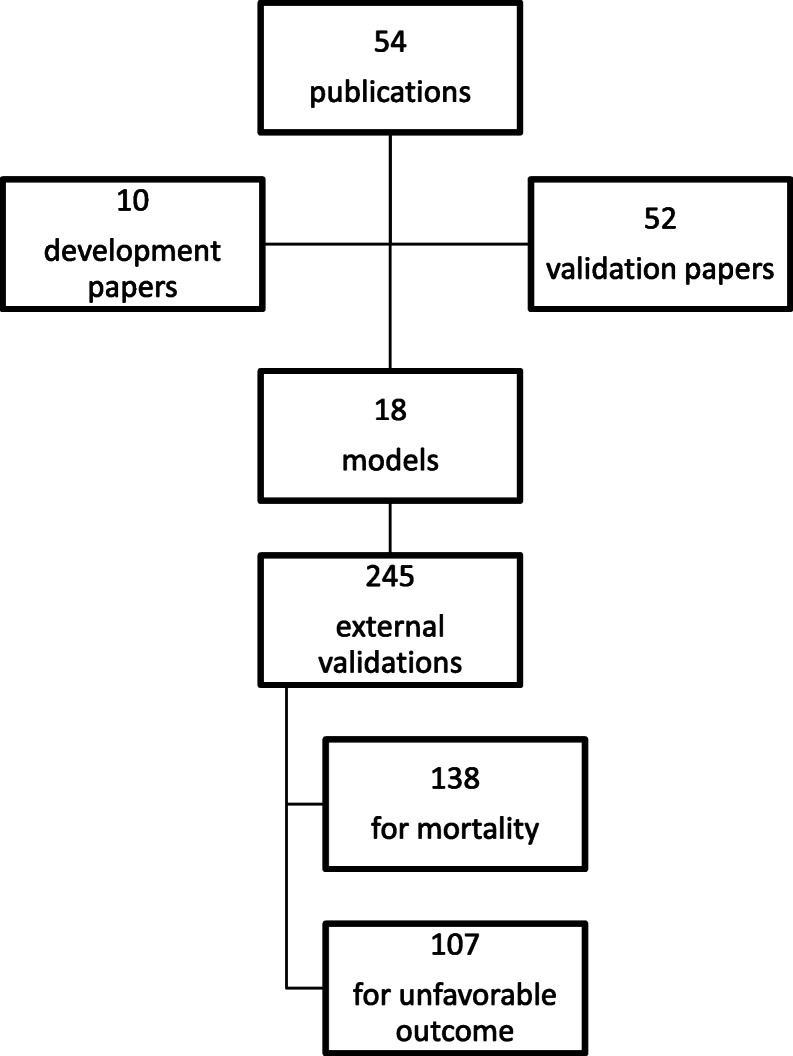
Flow diagram of included studies based on the systematic search

We included 54 publications comprising 18 multivariable regression models (Fig. 1). The publications include ten (10/55) model development papers, describing 18 models, and 52 (52/54) validation papers, describing 245 external validations. These 18 models were previously described by Dijkland et al. (2020), and no additional models were included based on the updated search strategy.

### Study characteristics

The 18 multivariable prognostic models predict mortality or unfavorable outcome at discharge or up to 12 months after hospital admission and were published between 1985 and 2021 (Supplementary Table 5). Four models (4/18; 22%) were developed in adult patients (aged > 14 years) who were admitted to the ICU [11–13], and fifteen models (14/18; 78%) were specifically developed in patients with TBI [9, 14–19]. Data for model development were collected through single or multi-center observational cohort studies, randomized controlled trials (RCTs), or pooled data derived from both cohort studies and RCTs. All studies, except for Yuan et al. [19], used prospective data.

Candidate predictors of outcome following TBI were collected at admission and typically included a combination of demographic, clinical, and radiology characteristics. The number of missing predictor and outcome data was not reported in three studies (3/10; 30%) (Supplementary Table 5 continued). Three studies (3/10; 30%) applied imputation methods for handling missing data. Seven studies (7/10; 70%) used a selection procedure, for instance stepwise selection, to reduce the number of predictors that were included in the final model.

Five studies (5/10; 50%) used an internal validation procedure (e.g., bootstrap validation procedure or cross-validation), whereas in the other five studies (5/10; 50%) the internal validation procedure was lacking or inefficient (split-sample procedure).

The AUCs at development ranged between 0.71 and 0.90 for the prediction of mortality, and between 0.65 and 0.90 for the prediction of unfavorable outcome. Of the nine development studies that described model performance in terms of calibration, three studies (3/9; 33%) exclusively reported the Hosmer-Lemeshow goodness-of-fit test and one (1/9; 11%) exclusively showed calibration graphically using a calibration plot, whereas five studies (5/9; 55%) reported both the Hosmer-Lemeshow goodness-of-fit test and a calibration plot.

### Methodological quality of model development studies

Methodological quality of model development studies was assessed in terms of applicability and risk of bias (RoB) with the PROBAST checklist (Table 1). Of the ten model development studies, eight (8/10; 80%) were judged high RoB (Table 2). In each case (8/8), the statistical analysis (analysis domain) resulted in a high RoB, due to insufficient sample size, suboptimal handling of missing data, and lack of or insufficient internal validation procedures (e.g., split-sample procedure). Four model development studies (4/10; 40%) were deemed high RoB in terms of applicability as these models were developed for patients admitted to the ICU and not strictly for patients following moderate and severe TBI. For most studies (9/10), the overall judgment on a short form based on the PROBAST, including 8/20 signaling questions, was consistent with the original PROBAST (Supplementary Table 6). Based on the short form, one study was identified as low RoB, but unclear RoB (CRASH models) on the original PROBAST, due to key information that was not reported.

**Table 2:** Overview of risk of bias, applicability, usability, and similarity in study design of development and validation studies

Model development studies (***N*** = 10 development studies)		
**Overall risk of bias of development studies**		
High	6	60%
Low	2	20%
Unclear	2	20%
**Applicability of development studies**		
High	3	30%
Low	7	70%
Unclear	0	0%
**Usability of models**		
*Research*		
Yes	4	40%
No	6	60%
*Clinical practice*		
Yes	9	90%
No	1	10%
**External validation studies (*N*= 245)**		
**Similarity in study design between development and validation cohorts**		
Similar	147	60%
Cohort to trial	26	11%
Trial to cohort	71	29%
NA	1	
**Relatedness**		
Related	35	14%
Moderately related	45	18%
Distantly related	164	67%
NA	1	

Risk of bias: risk of bias was assessed with the original PROBAST (Supplementary Table 3)

Usability: The model was deemed usable in research if the full model equation or sufficient information to extract the baseline risk (intercept) and individual predictor effects was reported, and usable in clinical practice if an alternative presentation of the model was included (e.g., a nomogram, score chart or web calculator)

Relatedness: To judge relatedness we created a relatedness rubric, aiming to capture various levels or relatedness by dividing the validation studies into three categories: “related,” “moderately related,” and “distantly related” (Supplementary Table 4)

### Usability

Just over half of the model development studies (6/10; 60%) provided the full model equations or sufficient information to extract the baseline risk (intercept) and individual predictor effects (regression coefficients). Most (8/10; 80%) studies included a presentation of the final prediction models, such as a nomogram or score chart, which makes implementation of the model in clinical practice more feasible (Table 2). Almost half of the studies (4/10; 40%) included insufficient information to externally validate the models (Table 2).

### External validation

The 18 prognostic models were externally validated 245 times (Supplementary Table 7). The IMPACT prognostic models were externally validated most extensively (127 times), followed by the CRASH models (56 times). Most (164/245, 67%) of the validation studies were judged “distantly related” (Table 2), indicating that the validation cohort substantially differed from the model development study in terms of inclusion criteria and/or outcome assessment. Furthermore, 45/245 (18%) of the validation studies were judged “moderately related,” as the models were validated in a different setting (e.g., country) than the model was originally developed in.

**Fig. 2:**
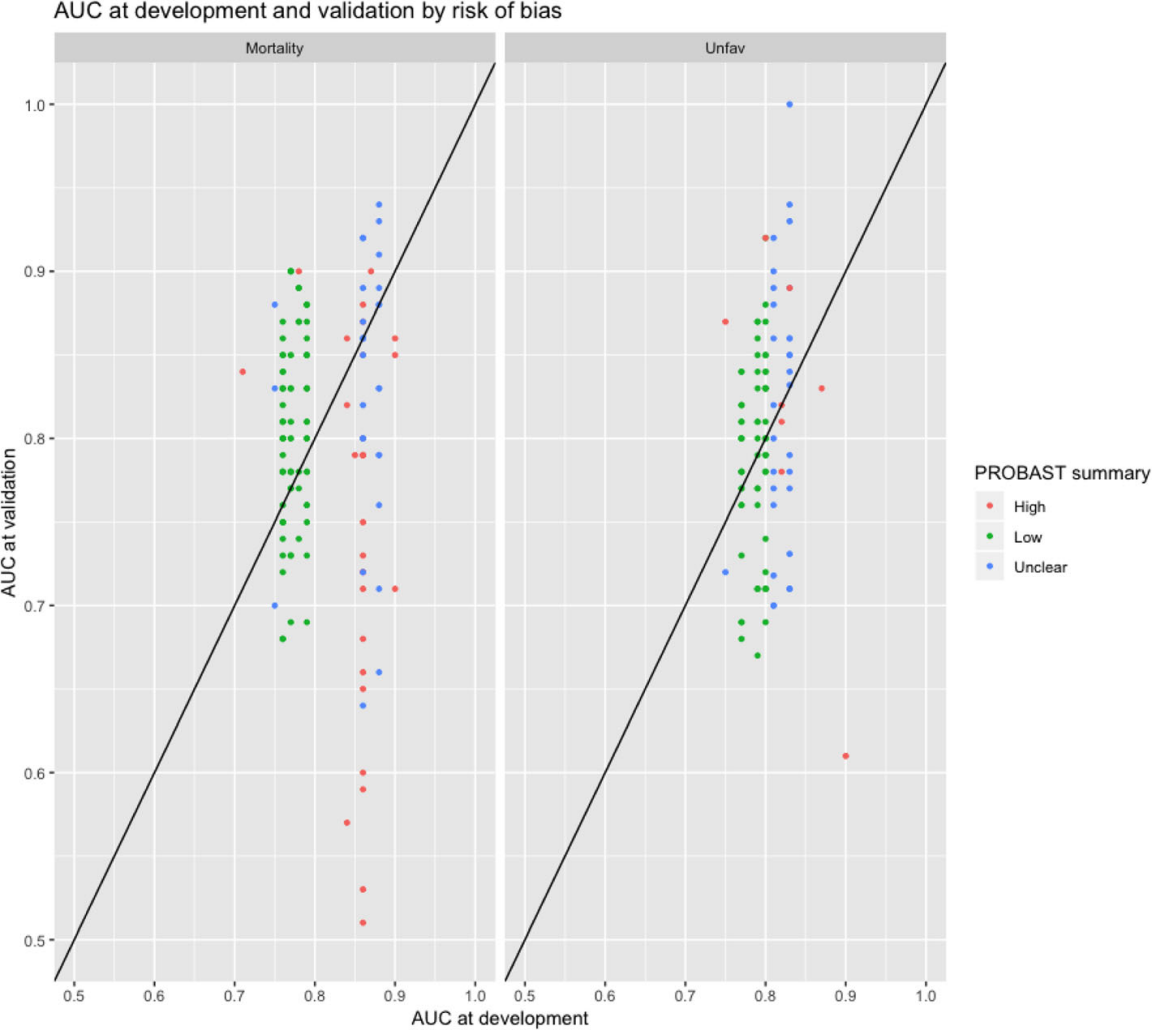
AUC of 18 models at development and in 242 validation studies by risk of bias assessed with the PROBAST

The discriminative ability of the models showed substantial variation (Supplementary Table 8; Fig. 2). Overall, the AUCs at external validation ranged between 0.47 and 0.94 for the prediction of mortality, and between 0.61 and 1.00 for the prediction of unfavorable outcome.

There was substantial variation in the agreement between observed and predicted probabilities. The reported calibration intercept ranged between − 1.27 and 0.93 for mortality, and between − 0.51 and 2.39 for the prediction of unfavorable outcome. The reported calibration slopes ranged between 0.72 and 2.3 for mortality and between 0.71 and 2.5 for unfavorable outcome (Supplementary Table 7).

### Relation between methodological quality and model performance

The difference between the AUC at development and validation was highly variable (Fig. 2). The median change in the discriminative ability in low RoB models was positive (*N* = 149 validation studies, dAUC 8%, [IQR − 4% to 21%]) compared to a negative median dAUC in high ROB models (*N* = 45 validation studies, dAUC − 18%, [IQR − 43% to − 2%]) (Table 3).

**Table 3:** The median AUC at development and external validation and the absolute and percentage change between development AUC and validation AUC stratified by risk of bias (RoB) of model development studies based on the original PROBAST

	N	Median AUC at development (***N*** = 10) [IQR]	Median AUC at external validation (***N*** = 245) [IQR]	Median delta AUC [IQR]	Median AUC change in percentage [IQR]
Low RoB	139	0.78 [0.77, 0.79]	0.80 [0.76, 0.84]	0.02 [− 0.01, 0.06]	8% [− 4, 21]
High RoB	45	0.86 [0.84, 0.86]	0.79 [0.69, 0.84]	− 0.06 [− 0.16, − 0.01]	− 18% [− 43, − 2]
Unclear RoB	61	0.83 [0.81, 0.86]	0.83 [0.77, 0.88]	0.00 [− 0.06, 0.04]	0.0% [− 19, 10]

Using the GEE, we found a larger average negative change in discrimination for high ROB models (− 32% (95% CI: − 48 to − 15) and unclear RoB models (− 13% (95% CI: − 16 to − 10)) compared to that seen in low RoB models (Table 4), while taking into account the correlation between validations of the same model and similarity in study design between the development and validation study. Models that were developed in a cohort and validated in a trial had an estimated change in discrimination of − 18% (95% CI: − 26 to − 10), whereas models that were developed in a trial and validated in a cohort had an estimated change in AUC of 0.4% (95% CI: − 3 to 4), compared to models that were developed and validated in data derived from a similar study design.

**Table 4:** Results of generalized estimated equations (GEE) for the percentage change in AUC between 10 development and 245 validation studies

	Percentage change in AUC (95% CI)
**Intercept**	9.5% (5.5, 13.4)
**Risk of bias (low)**	
High	− 31.7% (− 48.2, − 15.2)
Unclear	− 13.4% (− 16.4, − 10.3)
**Study design (similar)**	
Cohort to trial	− 18.5% (− 26.2, − 10.8)
Trial to cohort	0.19% (− 3.7, 4.1)

The generalized estimated equations (GEE) model includes a random intercept on model level (*N* = 18), risk of bias assessment (low, high, unclear based on the original PROBAST), and similarity in study design between the development and validation study (Similar, Cohort to trial, Trial to cohort) to estimate the percentage change in AUC between the development and validation studies. The intercept indicates the percentage change in AUC for low risk of bias models with a similar study design between the development and validation study

## Discussion

We examined the relation between methodological quality of prediction model development studies and performance at external validation for prognostic models predicting outcome of patients after moderate or severe traumatic brain injury (TBI). Of the ten included model development studies, two studies (four models) were found to have low risk of bias (RoB) and were applicable for patients after moderate and severe TBI. The other eight publications (fourteen models) showed “high” or “unclear” RoB and had limited usability or applicability for patients after moderate and severe TBI. At external validation, model performance is typically reduced [20]. However, our findings showed that, on average, the change in discriminative ability was positive in validations of “low” RoB models meaning that the models performed better at external validation. Conversely, the change in discriminative ability was negative for “high” RoB models, which means that the models performed worse at external validation. Methodological quality of model development studies was associated with discriminative ability at external validation, implying that poor methodological quality results in poorer model performance in new patients and settings. A recent large-scale validation study of a short form based on the PROBAST in the field of cardiovascular disease showed that high RoB was associated with poorer discrimination [10]. Our study confirms these findings for prognostic models in the field of TBI.

We critically appraised and assessed methodological quality of model development studies using the PROBAST [7]. Since its publication the PROBAST has, for instance, been applied in the field of rehabilitation [21], cardiology [10], and infectious diseases (COVID-19) [22]. Consistent with prior studies, the overall judgment on the 20 PROBAST questions was often “unclear” or “high” [21–24], due to key details that were not reported [5]. These findings emphasize the importance of adherence to reporting guidelines, such as the TRIPOD reporting guideline [25]. Additionally, the PROBAST checklist, which includes 20 items on participant selection, study design, predictors, outcome, and statistical analysis, can inform investigators on what should be reported in prognostic model studies. A short form based on the PROBAST, consisting of 8/20 items, was recently validated and could distinguish well between high and low RoB [10]. In our study, the overall judgment on the short form was consistent with the original PROBAST for almost all studies.

A prior study reported that the majority of prediction studies in high impact journals did not follow methodological recommendations based on reporting statements, checklists, and quality assessment tools [26]. Similarly, in most model development studies included in our study, the statistical analyses were suboptimal due to insufficient sample size, suboptimal handling of missing data, stepwise selection procedures, and lack of or insufficient internal validation procedures, resulting in a high RoB. Consistent with prior studies that have critically appraised model development studies in TBI, internal validation studies of models developed before 2005 were often lacking or inefficient [3, 4]. In contrast, models that were developed more recently, between 2005 and 2021, did more often include an internal validation procedure. In recent years, the importance of internal validation has been stressed [27, 28] and internal validation procedures are accessible through free statistical software such as R [29]. These developments may have resulted in a higher uptake of these practices.

External validation aims to examine how the model performs in new patients from different settings [30]. This may relate to model performance in patients from different regions or countries (geographical validation), or in patients that differ from the derivation cohort on a characteristic (domain validation) [2]. External validation, preferably across a range of settings, is required before clinical application of a model can be recommended. Varying levels of relatedness between the development and validation study are expected. We used a relatedness rubric to define the consistency between development and validation studies, using three categories: “related,” “moderately related,” and “distantly related” [13]. Most of the validation studies differed substantially from the model development study in terms of inclusion criteria and/or outcome assessment, and were judged “distantly related.”

Differences in case-mix (distribution of patient characteristics) might arise from various levels of relatedness between the development and validation study and differences in study design between the development and validation study. Case-mix differences typically affect the observed change in discrimination [31]. Differences in case-mix are expected between observational cohort studies and RCTs, with cohort studies being more heterogeneous. We found that similarity in study design between the development and validation study was associated with the observed change in discriminative ability. For instance, models that were developed in a cohort and validated in a trial had worse discriminative ability at external validation, whereas models that were developed in a trial and validated in a cohort had better discriminative ability at external validation, compared to models that were developed and validated in data derived from a similar study design. These findings reflect larger case-mix heterogeneity in cohorts versus trials. Differences in case-mix can be measured through the model based concordance (c) statistic (mbc) [32], which provides insight into the influence of case-mix heterogeneity on the discriminative ability. In our study, the mbc was reported in only two of the validation studies published after its introduction in 2016 [33, 34].

Prior systematic reviews found that calibration, the agreement between observed and predicted outcomes, is described less often than discrimination [5, 26, 35]. Similarly, a number of the external validation studies did not assess model performance in terms of calibration. When reported, calibration was assessed with the Hosmer-Lemeshow goodness-of-fit test [36] or shown graphically with a calibration plot. The Hosmer-Lemeshow statistic has poor power to detect various violations of model assumptions [37]. Although broadly used as a measure of calibration in validation studies, this statistic is not recommended for this purpose [38]. To be able to compare model performance between validation studies, reporting the calibration intercept and slope is preferred. Dijkland et al. [6] concluded that the calibration of models for moderate and severe TBI was highly variable, reflecting heterogeneity in reliability of predictions, which motivates continuous validation and updating if clinical implementation is pursued.

### Strengths and limitations

The key strength of this study is that a risk of bias assessment (PROBAST) was related to model performance in external validation studies. Although the “Explanation and Elaboration” form provides extensive instructions for the scoring of PROBAST, many items are open for interpretation and the overall judgment is dependent on decisions that are made throughout the reviewing process. For instance, to determine if there was a reasonable number of outcome events relative to the number of predictors, we used EPP ≥ 10, which is widely adopted in prediction modeling studies as the minimal guideline criterion for binary logistic regression analysis. However, more recently, authors have suggested higher EPP’s of at least 20 and criteria that consider the outcome prevalence, overall model performance, and predictor distributions to determine the sample size required [39]. In our study, two of the twelve model development papers were assessed by a third independent reviewer (LW) (Cohen’s kappa = 0.64). In each case, the disagreement between the reviewers were “no information” versus “(probably) yes,” and they did not influence the overall RoB score.

We included 18 prognostic models for functional outcome following moderate and severe TBI that were externally validated at least once. Although the assessment of model performance in new patients and settings is crucial, external validation is often lacking [20]. Therefore, we could include only a limited number of models. In our study, we decided to examine the association between methodological quality and performance in terms of discrimination and not calibration for several reasons. First, calibration is less often described than discrimination. The calibration at external validation using the calibration intercept and slope was reported for only 8 of 18 models. Second, different measures (e.g., Hosmer-Lemeshow goodness-of-fit test, calibration plot, calibration intercept (calibration-in-the-large) and slope) are used to assess calibration, which makes it more difficult to compare calibration between validation studies. These different calibration measures, such as the calibration intercept and slope, are likely to be affected differently by methodological quality of the development study. Third, apart from methodological quality of the development study, calibration is likely influenced by relatedness between the development and validation study. Thus, calibration can be highly variable between external validation studies because of differences in setting and patient characteristics. For instance, it can be strongly influenced by differences in outcome rates between development and validation, beyond what is predicted by the model. Furthermore, consistent with prior studies, there was low variability in the PROBAST overall judgments as well as the relatedness assessment. Because of the limited sample size and low variability, additional variables that might have an effect on the observed change in discrimination (e.g., relatedness) were not included in the GEE. Other variables (e.g., usability and applicability) were not included in the GEE as they were not expected to have an effect on the observed change in discrimination. The models with low RoB, the Hukkelhoven model and IMPACT models [9, 15], were externally validated more frequently than the models classified as high RoB. This implies that the number of external validations might be related to methodological quality of the model development study. Apart from low RoB, these models were also presented with sufficient information to be externally validated. Our results are limited in terms of number of models, but confirm findings from a larger study, which showed that most published prediction models are at high RoB and that high RoB is associated with poorer discrimination. A previous study by Venema et al. (2021) included 556 prediction models for cardiovascular disease, with 1147 validations from the Tufts Predictive Analytics and Comparative Effectiveness (PACE) CPM Registry [10]. Venema et al. also corrected for other factors that could be related to the difference in model performance between development and external validation, including overlap in authors between development and validation study, sample size at validation, and years between the development and validation study. In our study, we did not assess methodological quality of the validation studies, which could also influence the difference in model performance between the development and validation study. Future research should further explore the association between methodological quality of external validation studies and model performance.

## Conclusion

Higher methodological quality of model development studies is associated with better model performance at external validation in the field of TBI. Our findings support the importance of adherence to methodological principles at model development and following guidelines for reporting of prediction modeling studies.

## Supplementary Information

**Additional file 1: Supplementary Table 1**: Inclusion criteria of systematic search (Adapted from Dijkland et al., (2020)). **Supplementary Table 2**: Search strategy (Dijkland et al., 2019). **Supplementary Table 3**: Prediction model Risk Of Bias Assessment Tool (PROBAST) items and guidelines for reviewers. **Supplementary Table 4**: Relatedness rubric. **Supplementary Table 5**: Data extraction of 10 model development studies describing 18 prediction models. **Supplementary Table 6**: Methodological quality of model development studies for outcome following moderate and severe traumatic brain injury in terms of Applicability and Risk of Bias assessed with a short form based on the PROBAST. **Supplementary Table 7**: Data extraction of validation studies of 18 prediction models for outcome following moderate and severe traumatic brain injury. **Supplementary Table 8**: Median AUC and IQR at development and validation for each model.

## Data Availability

Not applicable.

## Abbreviations

AUC: Area under the operating receiver curve
GEE: Generalized estimation equations
Mbc: Model based concordance (c) statistic
PROBAST: Prediction model Risk Of Bias Assessment Tool
RCT(s): Randomized controlled trial(s)
RoB: Risk of bias
TBI: Traumatic brain injury
