# Supplementary material for: Does poor methodological quality of prediction modeling studies translate to poor model performance? An illustration in traumatic brain injury
Source: Diagn Progn Res. 2022 May 5;6:8. doi: 10.1186/s41512-022-00122-0 (PMC9068255; doi:10.1186/s41512-022-00122-0)
Supplement: Supplementary file 1 — Additional file 1: Supplementary Table 1: Inclusion criteria of systematic search (Adapted from Dijkland et al., (2020)). Supplementary Table 2: Search strategy (Dijkland et al., 2019). Supplementary Table 3: Prediction model Risk Of Bias Assessment Tool (PROBAST) items and guidelines for reviewers. Supplementary Table 4: Relatedness rubric. Supplementary Table 5: Data extraction of 10 model development studies describing 18 prediction models. Supplementary Table 6: Methodological quality of model development studies for outcome following moderate and severe traumatic brain injury in terms of Applicability and Risk of Bias assessed with a short form based on the PROBAST. Supplementary Table 7: Data extraction of validation studies of 18 prediction models for outcome following moderate and severe traumatic brain injury. Supplementary Table 8: Median AUC and IQR at development and validation for each model. [file 41512_2022_122_MOESM1_ESM.docx]

**Supplementary materials**

| **Supplementary Table 1:** Inclusion criteria of systematic search (Adapted from Dijkland et al., (2020)) |
| --- |
| **Inclusion** |
| *Studies* |
| English language |
| Published between January 2006 and June 2021^a^ |
| Development, update or extension of prognostic model^a^ |
| Development study has to report the discriminative ability of the model^a^ |
| *Prediction models* |
| Multivariable prediction models |
| Prognostic prediction models |
| Based on admission characteristics |
| Validated at least once in the same, or a separate publication^a^ |
| *Population* |
| Traumatic brain injury |
| Glasgow Coma Scale ≤ 12 |
| Age ≥ 14 years |
| *Outcome* |
| Functional outcome |
| ^a^ Adaptations made from the original systematic search by Dijkland et al. (2020) |

| **Supplementary Table 2:** *Search strategy (Dijkland et al., 2019)* | |
| --- | --- |
| Database | Search |
| Embase.com | ('traumatic brain injury'/exp OR 'brain injury'/de OR 'head injury'/de OR 'acquired brain injury'/de OR ('nervous system injury'/de AND brain/exp) OR (((trauma* OR injur* OR damage*) NEAR/3 (brain* OR cerebral* OR head OR cranial* OR intracranial*)) OR tbi):ab,ti) AND (((model/de OR 'mathematical model'/de OR 'disease model'/de) AND ('prognosis'/de OR 'prediction'/de OR 'mortality'/de OR 'survival'/de OR fatality/de OR 'convalescence'/de OR 'predictive validity'/de )) OR 'nomogram'/de OR (((prognos* OR predict* OR mortal* OR convalescen* OR recover* OR surviv* OR fatal*) NEAR/6 (model*)) OR nomogram*):ab,ti) NOT ([animals]/lim NOT [humans]/lim) NOT ([Conference Abstract]/lim OR [Letter]/lim OR [Note]/lim OR [Editorial]/lim) AND [english]/lim |
| Medline Ovid | ("Brain Injuries"/ OR exp "Brain Hemorrhage, Traumatic"/ OR "Craniocerebral Trauma"/ OR "Head Injuries, Closed"/ OR "Head Injuries, Penetrating"/ OR ("Trauma, Nervous System"/ AND exp brain/) OR (((trauma* OR injur* OR damage*) ADJ3 (brain* OR cerebral* OR head OR cranial* OR intracranial*)) OR tbi).ab,ti.) AND (((exp "Models, Statistical"/ OR exp "Models, Theoretical"/) AND ("prognosis"/ OR exp "mortality"/ OR "mortality".xs. OR survival/ OR "Fatal Outcome"/ OR "Convalescence"/ )) OR "Nomograms"/ OR (((prognos* OR predict* OR mortal* OR convalescen* OR recover* OR surviv* OR fatal*) ADJ6 (model*)) OR nomogram*).ab,ti.) NOT (exp animals/ NOT humans/) NOT (letter OR news OR comment OR editorial OR congresses OR abstracts).pt. AND english.la. |
| PsycINFO Ovid | ("Traumatic Brain Injury"/ OR exp "Brain Damage"/ OR "Head Injuries"/ OR (((trauma* OR injur* OR damage*) ADJ3 (brain* OR cerebral* OR head OR cranial* OR intracranial*)) OR tbi).ab,ti.) AND ((("Models"/ ) AND ("prognosis"/ OR exp "Death and Dying"/ OR "Mortality Rate"/ )) OR "Nomograms"/ OR (((prognos* OR predict* OR mortal* OR convalescen* OR recover* OR surviv* OR fatal*) ADJ6 (model*)) OR nomogram*).ab,ti.) NOT (exp animals/ NOT humans/) NOT (letter OR news OR comment OR editorial OR congresses OR abstracts OR books).pt. AND english.la. |
| Cochrane central | ((((trauma* OR injur* OR damage*) NEAR/3 (brain* OR cerebral* OR head OR cranial* OR intracranial*)) OR tbi):ab,ti) AND ((((prognos* OR predict* OR mortal* OR convalescen* OR recover* OR surviv* OR fatal*) NEAR/6 (model*)) OR nomogram*):ab,ti) |
| Web of Science | TS=(((((trauma* OR injur* OR damage*) NEAR/2 (brain* OR cerebral* OR head OR cranial* OR intracranial*)) OR tbi)) AND ((((prognos* OR predict* OR mortal* OR convalescen* OR recover* OR surviv* OR fatal*) NEAR/5 (model*)) OR nomogram*)) NOT ((animal* OR rat OR rats OR mouse OR mice OR  murine OR cat OR cats OR feline OR dog OR dogs OR canine OR sheep OR ovine OR cow OR bovine OR cattle OR horse OR equin* OR pig OR swine OR porcine OR monkey* OR primate* OR gerbil* OR rabbit* OR rodent*) NOT (human* OR patient*))) AND DT=(article) AND LA=(english) |
| Google scholar | "brain\|cerebral\|head\|cranial\|intracranial trauma\|injury\|injuries\|damage"\|tbi "prognosis\|prognostic\|predictive\|survival model\|models"\|" model\|models*mortality\|convalescence\|recovery\|fatality\|fatal"\|nomogram\|nomograms |

| **Supplementary Table 3:** *Prediction model Risk Of Bias Assessment Tool (PROBAST)* *items and guidelines for reviewers* | | |
| --- | --- | --- |
| **Item** | **Question** | **Guidelines for reviewers** |
| **Participants** | | |
| 1.1 | Were appropriate data sources used, e.g. cohort, RCT or nested case-control study data? | Yes: Cohort, RCT, nested case-control  No: -  NI: Source of data not reported |
| 1.2 | Were all inclusions and exclusions of participants appropriate? | Yes: If exclusion was reasonable (e.g. patients with dementia or neurological conditions)  No: If exclusion was unreasonable (e.g. patients that were not recovered at the end of the study)  NI: Inclusion and exclusion criteria not reported |
| *RoB* | Participants (Low, High, Unclear) | Low: all items scored as ‘yes’  High: at least one item scored as ‘no’  Unclear: at least one item scored as ‘NI’ |
| *Applicability* | Concerns for applicability (Low, High, Unclear) | Low: Adults, moderate and/or severe TBI, based on GCS ≤12, all strata  High: none TBI patients (e.g. trauma or ICU patients) |
| **Predictors** | | |
| 2.1 | Were predictors defined and assessed in a similar way for all participants? | Yes: All predictors are defined and assessed in a similar way for all participants.  No: Predictors are defined and assessed differently for patients. However, we decided not to judge as 'High' RoB only based on 2.1. For instance, depending on the predictor, it was appropriate if predictor was assessed clinically for some patients and based on clinical suspicion for other patients (e.g. alcohol intoxication).  NI: NA, If no information was provided we assumed that predictors were defined and assessed in a similar way for all participants. |
| 2.2 | Were predictor assessments made without knowledge of outcome data? | Yes: If the study was prospective we assumed that the predictor assessments were made without knowledge of outcome data.  No: -  NI: If the study was retrospective and no information was provided on blinding for outcome data when assessing predictors.  PROBAST E&E: In prognostic studies, this signaling question should thus be answered as NI. However, the domain can still be rated as low RoB in the overall RoB assessment because if predictors were measured and reported  a long time before the outcome occurred, their measurement can be inferred to be “blinded to the outcome.” |
| 2.3 | Are all predictors available at the time the model is intended to be used? | Yes: Because the models included baseline variables for prediction of functional outcome following TBI we assumed that they were available at the time the model is intended to be used |
| *RoB* | Risk of bias: Predictors (Low, High, Unclear) | Low: all items scored as ‘yes’, also if 2.1 scored as ‘pn’, or 2.2 scored as ‘ni’.  High: if 2.3 was scored as ‘no’. When only 2.1 was ‘pn’ or 2.2 ‘ni' overall RoB is not judged as 'high'. When 2.2 is 'ni' overall RoB can still be 'Low', if predictors were reported a long time before the outcome occured.  Unclear: at least one item scored as ‘NI’, expect for 2.2. |
| *Applicability* | Concerns for applicability (Low, High, Unclear) | Low: Admission characteristics |
| **Outcome** | | |
| 3.1^a^ | Was the outcome determined appropriately? | Yes: GOS(-E) or mortality  No:  NI: Not reported how outcome was determined. For mortality it is not necessary to report how the outcome was determined |
| 3.2^a^ | Was a pre-specified or standard outcome definition used? | Yes: Glasgow Outcome Scale (-Extended) (GOSE) or mortality  NI: Not reported. For mortality the outcome definition is typically not defined, but is not considered necessary, thus 3.2 was rated as ‘py’ when the outcome was mortality or survival |
| 3.3 | Were predictors excluded from the outcome definition? | Yes: if predictors did not overlap with outcome |
| 3.4^a^ | Was the outcome defined and determined in a similar way for all participants? | Yes: If outcome was defined and determined in a similar way for all patients. If the GOS(E) was completed by some patients via telephone, filled in via the questionnaire or via an interview this was scored as ‘yes’.  No: If outcome was not defined and determined in a similar way for all patients.  NI: NA, If no information was provided we assumed that outcome was defined and determined in a similar way for all participants. |
| 3.5 | Was the outcome determined without knowledge of predictor information? | Yes: Although typically there is no information provided we assumed that outcome was determined without knowledge of predictor information |
| 3.6 | Was the time interval between predictor assessment and outcome determination appropriate? | Yes: Because the models included admission characteristics for prediction of functional outcome following TBI we assumed that the time interval was appropriate  No: -  NI: if no information about timing of predictor and outcome assessment |
| *RoB* | Risk of bias: Outcome (Low, High, Unclear) | Low: all items scored as ‘yes’  High: at least one item scored as ‘no’  Unclear: at least one item scored as ‘NI’ |
| *Applicability* | Concerns for applicability (Low, High, Unclear) | Mortality, unfavourable outcome, good recovery based on GOS(E).  Mortality can be based on hospital records. |
| **Analysis** | | |
| 4.1^a^ | Were there a reasonable number of participants with the outcome? | Yes: EPP ≥ 10  No: EPP ≥ 10  NI: if sample size or number of predictors were not available  The number of events in the smallest group was divided by the total degrees of freedom used during the whole modelling process. The total degrees of freedom was based on the number of variables (continuous variables) or categories (categorical variables) in the model (Events Per Parameter (EPP)). All candidate predictors were considered as part of the modelling process, including those not selected for the multivariable model based on univariable regression analysis or selection procedures. We assumed a reasonable number of outcome events when EPP ≥ 10. |
| 4.2^a^ | Were continuous and categorical predictors handled appropriately? | Check for linearity adjustments.  Yes: Continuous predictors are not converted into >2 categories (ie, dichotomized or categorized), or continuous predictors are converted into >2 categories based on pre-specified or widely accepted cut points, which should be explicitly mentioned in the paper, or continuous predictors are examined for non-linearity (e.g. with exponential, fractional polynomial or spline function), or categorical predictor groups are defined using a pre-specified or widely accepted method (i.e. not based on the own data).  No: Continuous predictors are converted into >2 categories or categorical predictor groups definitions are based on the cut point or threshold in the current dataset.  NI: No information on how predictors were included in the model. |
| 4.3 | Were all enrolled participants included in the analysis? | Yes: if yes.  No: if subgroups of patients were excluded from the analysis.  NI: if not reported, we assumed ‘py’. |
| 4.4^a^ | Were participants with missing data handled appropriately? | Yes: The study explicitly reports that there are no missing values of predictors or outcomes, or if Single or multiple imputation was performed. Complete case analysis was judged as appropriately if low % (<10%) missing and clear justification was declared for complete case analysis.  No: complete case analysis with high % missing and no justification was declared for complete case analysis.  NI:NA, If no information was provided on how missing data was handled we assumed complete case analysis without justification, thus scored as ‘No’. We did not require subgroup analysis for participants with an without missing data.  PROBAST E&E: “No information was provided to confirm that complete-case analysis was a valid approach—that is, whether the included and excluded participants were similar such that the included participants approximated a completely random subset of the original study sample. Accordingly, this signaling question should be answered as N for development.” |
| 4.5^a^ | Was selection of predictors based on univariable analysis avoided? | Yes: No univariable analysis  No: Univariable analysis was performed to select predictors that were included in the multivariable analysis  NI: NA; assume that univarible analysis were not conducted if there was no information, thus score as ‘Yes’ |
| 4.6 | Were complexities in the data (e.g. censoring, competing risks, sampling of controls) accounted for appropriately? | Question is only relevant for case-control, nested case control and cox regression. Therefore, this items was scored as ‘NA’ for all included studies. |
| 4.7 | Were relevant model performance measures evaluated appropriately? | Discrimination: ROC/AUC, C-statistic.  Calibration: Calibration plot, and optionally Hosmer and Lemeshow goodness of fit test.  Only discrimination was required for development studies.  Yes: AUC or C-statistic was reported  No: No AUC or C-statistic was reported  NI: NA; assume that model performance was not evaluated appropriately if there was no information on discrimination, thus score as ‘No’ |
| 4.8^a^ | Was model overfitting and optimism in model performance accounted for? | Yes: Bootstrap procedure or cross-validation  No: split-sample or no procedure to correct for model overfitting or optimism  NI: NA, if no information was included we assumed that there was no procedure to account for overfitting and optimism, thus was answered ‘No’  Preferably we would check if the bootstrapping or cross-validation did include all model development procedures including any variable selection, and check if adjustment of the prediction model performance measures have been applied (ie, “internally validated AUC”). However, this information is typically not described. |
| 4.9 | Do predictors and their assigned weights in the final model correspond to the results from the reported multivariable analysis? | Yes: If results from multivariable analysis and final model formula correspond. However, if only results from multivariable analysis were reported or only the formula this was rated as ‘py’.  NI: If intercept and coefficients nor a model formula was reported. Also score ‘NI’ if only web-based calculator or score-chart is available without including the formula. |
| *RoB* | Risk of bias: Analysis (Low, High, Unclear) | Low: all items scored as ‘Yes’  High: at least one item scored as ‘No’  Unclear: at least one item scored as ‘NI’ |
| **Overall judgment** | | |
| Overall judgement of RoB | Risk of bias: Overall (Low, High, Unclear) | Low: all items scored as ‘Low  High: at least one item scored as ‘High  Unclear: at least one item scored as ‘Unclear |
| ^a^ PROBAST items included in a short form | | |

| **Supplementary Table 4:** *Relatedness rubric* | | |
| --- | --- | --- |
| **Criteria** | | |
| A. Setting | Country  Stratum (Emergency department, Hospital ward, Intensive Care Unit) | |
| B. Inclusion criteria | Patient’s eligibility | |
| C. Outcome | Outcome  Timing | |
| **Overall judgement** | | |
|  | Related | No differences between development and validation study on A, B and C |
|  | Moderately related | Differences between development and validation study on criteria A alone |
|  | Distantly related | Differences between development and validation study on criteria B or C |

| **Supplementary Table 5:** *Data extraction of 10 model development studies describing 18 prediction models* | | | | | | | | |
| --- | --- | --- | --- | --- | --- | --- | --- | --- |
|  |  | **Source of data** | | | **Participants** | **Outcome** | | **Sample size** |
| **First author;**  **Year of publication** | **Model** | **Period of study** | **Data collection** | **Study design** | **Inclusion criteria** | **Outcome; Measure** | **Time of outcome** | **Number of total patients; N with outcome of interest** |
| Knaus;  1985 | APACHE II | 1979-1982 | Prospective | Multi-center observational cohort study | Patients admitted to the ICU | Mortality | In hospital | Total: 5815;  Mortality: 993 |
| Le Gall;  1993 | SAPS II | September 1991 - February 1992 | Prospective | Multi-center observational cohort | >18 years; ICU | Mortality | Discharge | Total: 8548;  Mortality: 1836 |
| Lemeshow; 1993 | MPM II:   - MPM0 - MPM24 | Data Set I April 1989- July 1990. Data Set II, September 1991-December 1991 | Prospective | Multi-center observational cohort | >18 years; ICU | Mortality | In hospital | Total: 12610;  Mortality: 2632 |
| Signorini; 1999 | Signorini | January 1989-July 1991 | Prospective | Single-center cohort study | ≥14 years; GCS ≤12, or GCS 13-15 with ISS ≥16 or more | Survival/Mortality | Twelve months | Total: 372;  Survival: 278;  Mortality: 87 |
| Hukkelhoven; 2005 | Hukkelhoven model | 1992-1994 (Tirilazad international), 1991-1994 (Tirilazad North America) | Prospective | Two multi-center trials | 15–65 years; Severe (GCS 3–8) or moderate (GCS 9–12) closed TBI | Mortality, Unfavorable outcome;  Glasgow Outcome Scale | Six months | Total: 2269 (mortality) and 2137 (unfavorable outcome) patients;  Mortality: 500,  Unfavorable outcome: 862 |
| Maas;  2005 | Rotterdam CT score | 1992-1994 (Tirilazad international), 1991-1994 (Tirilazad North America) | Prospective | Two multi-center clinical trials | 15-65 years; severe (GCS 3–8) or moderate (GCS, 9–12) closed TBI | Mortality; Glasgow Outcome Scale | Six months | Total: 2249;  Mortality: 491 |
| Perel;  2008 | CRASH:   - Core - CT | April 1999 - December 2000 | Prospective | Multi-center clinical trial | ≥16 years; GCS ≤ 14; Within eight hours of injury | Mortality, Unfavorable outcome; Glasgow Outcome Scale | 14 days and six months | Total: 10.008;  Mortality: 2396; Unfavorable outcome: 4082 |
| Steyerberg;  2008 | IMPACT:   - Core - Extended - Lab | 1984-1997 | Prospective | Eight randomized controlled trials, three observational cohort studies | ≥ 14 years; moderate and severe TBI (GCS ≤ 12) | Mortality, Unfavorable outcome; Glasgow outcome scale | Six months | Total: 8.509;  8.509 Core,  6.999 extended,  3.554 lab;  Mortality: 2396; Unfavorable outcome: 4082 |
| Jacobs;  2012 | Nijmegen:   - Clinical - Combination - CT | January 1998-January 2006 | Prospective | Single-center observational cohort | > 16 years; Moderate (GCS 9-12) and severe (GCS ≤ 8) TBI; ED | Mortality, Unfavorable outcome, Unfavorable outcome excluding death; Glasgow Outcome Scale | Six months | Total: 605 and 567; Mortality: 251,  Unfavorable outcome: 328, Unfavorable outcome excl. deaths: 77 |
| Yuan;  2012 | Yuan:   - Model A - Model B - Model C | January 2007-December 2009 | Retrospective | Single-center cohort study | ≥ 18 years; Glasgow Coma Scores ≤ 12, for whom at least one computed tomography (CT) scan and laboratory examination was performed after injury | Mortality, Favorable outcome; Glasgow Outcome Scale | 30 days and six months | Total: 1016;  Mortality: 265;  Unfavorable outcome: 432; Favorable outcome: 584 |
| APACHE II, Acute Physiology and Chronic Health Evaluation II; IMPACT, International Mission for Prognosis and Analysis of Clinical Trials; | | | | | | | | |

| **Supplementary Table 5 continued:** *Data extraction of 10 model development studies describing 18 prediction models* | | | | | | | | | |
| --- | --- | --- | --- | --- | --- | --- | --- | --- | --- |
|  |  | **Predictors** | **Missing data** | | **Model development** | | **Model performance** | | **Results** |
| **First author;**  **Year of publication** | **Model** | **Number of predictors (candidate predictors); Number of regression coefficients; Time of assessment** | **Missing data: Predictors;**  **Outcomes** | **Handling of missing data** | **Modeling method; Selection procedure** | **Internal validation procedure** | **Discrimination** | **Calibration** | **Full model equation(s) and alternative model presentation** |
| Knaus;  1985 | APACHE II | 9 (of 34);  15 regression coefficients;  Admission | Predictors: 756 missing, 13% of the 5815 ICU patients had missing predictor values;  Outcome: 786 missing, Postcoranary artery bypass graft excluded | Complete case analysis | Logistic regression analysis; Selection based on clinical judgement | Split-sample procedure | C-statistic | Calibration plot | Full model equations |
| Le Gall;  1993 | SAPS II | 14 (of 37);  17 regression coefficients;  First 24h in ICU | Not reported | Complete case analysis | Logistic regression analysis; Univariable analysis (p-value not reported), Multivariable analysis based on goodness of fit improvement | Split-sample procedure | C-statistic | Hosmer-Lemeshow test | Full model equations; Score chart |
| Lemeshow;  1993 | MPM II:   - MPM0 - MPM24 | 14 (of 22);  14 regression coefficients in MPM0, 13 in MPM24; Admission | Not reported | Complete case analysis | Logistic regression analysis;  Univariable analysis (p<.1) and at least 2% of the population exhibited that factor, Multivariable analysis with backward stepwise selection (p<.05), Variables were eliminated when it improved calibration while not significantly affecting discrimination | No internal validation procedure | C-statistic | Hosmer-Lemeshow test | Full model equations |
| Signorini;  1999 | Signorini | 8 (of 8);  10 regression coefficients in final model;  Admission | Predictors: GCS 3, pupil reactivity 102, CT diagnosis 74, alcohol consumed 62 observations missing; Outcome: 7 patients missing | Complete case analysis | Logistic regression analysis; Forward selection | No internal validation procedure | C-statistic | Hosmer-lemeshow test, Calibration plot | Full model equations; Nomogram |
| Hukkelhoven;  2005 | Hukkelhoven model | 9 (of 9);  15 regression coefficients in final model; First hours after injury and CT within 4 hours after injury | Predictors: Pupillary reactivity 70 and 211, hypoxia 130 and 133, hypotension 30 and 31, CT classification 8 and 12, traumatic subarachnoid hemorrhage 22 and 24. Outcome: 33 and 87 missing. | Imputation | Logistic regression analysis; Backward selection (p <.20) | Bootstrap validation | C-statistic | Hosmer-Lemeshow test; Calibration plot | Full model equations; Score chart |
| Maas;  2005 | Rotterdam CT score | 10 (of 10); 13 regression coefficients in final model;  CT scans were performed within 4h after injury | Predictors: Traumatic SAH 42, Intraventricular blood 30, Midline shift 38, Lesion 38 observations; Outcome: no missingness | Single imputation | Logistic regression analysis; Recursive portioning (CART) | Bootstrap validation | C-statistic | None | Full model equations; Score chart |
| Perel;  2008 | CRASH:   - Core - CT | 9 (of 13); 5 regression coefficients in the basic model, 10 in the CT model; admission | Not reported | Complete case analysis | Logistic regression analysis; Selection based on p-value* | Bootstrap validation | C-statistic | Hosmer-Lemeshow test, Calibration plot | Web-based calculator |
| Steyerberg;  2008 | IMPACT:   - Core - Extended - Lab | 17 (of 26); 8 regression coefficients in core model, 15 in extended model, 17 in lab model; Admission | Predictors: glucose 167, Hb 132, pupillary reactivity 1383 observations missing;  Outcomes: 1611 Glasgow Outcome Scores missing | Multiple imputation | Logistic regression analysis | Cross-validation | C-statistic | Hosmer-lemeshow test, Calibration plot | Full model equations online; Score chart |
| Jacobs;  2012 | Nijmegen:   - Clinical - Combination - CT | 14 (14); 8 regression coefficients in clinical model, 9 in combination model and 6 in CT model;  Clinical  Admission. Initial CT-scans within 24h after injury | Predictors:  Demographic and clinical data were missing in <0.5 % of patients. <4 % of all individual CT characteristics was missing. Missing not reported per predictor; Outcome: 95 missing | Complete case analysis | Logistic regression analysis; Univariable selection of significant predictors (p<.01), Forward selection (p<.01) | No internal validation procedure | C-statistic | Hosmer-Lemeshow test | Full model equations; Web-based calculator |
| Yuan;  2012 | Yuan:   - Model A - Model B - Model C | 10 (of 14); 8 regression coefficients in Model A, 13 in Model B, 17 in Model C, Admission and CT within 6h | Predictors: ICP 227 (22%) missing. | Complete case analysis | Logistic regression analysis | Bootstrap validation | C-statistic | Hosmer-lemeshow test, Calibration plot | Web-based calculator |
| Complete case analysis was assumed if method for handling missing data was not reported  *reported elsewhere | | | | | | | | | |

| **Supplementary Table 6:** *Methodological quality of model development studies for outcome following moderate and severe traumatic brain injury in terms of Applicability and Risk of Bias assessed with a short form based on the PROBAST* | | | | | | | | | |
| --- | --- | --- | --- | --- | --- | --- | --- | --- | --- |
| **Study** | **Models** | **Risk of bias based on a short form based on the PROBAST** | | | | | | | |
|  |  | Outcome assessment | Events per variable | Continuous predictors | Missing data | Univariable analysis | Correction for overfitting/optimism | Overall ROB short form for the PROBAST | Overall  ROB original PROBAST |
| PROBAST items | | 3.1; 3.2; 3.4 | 4.1 | 4.2 | 4.4 | 4.5 | 4.8 |  |  |
| Knaus | APACHE II | 0 | 0 | U | 1 | 0 | 1 | H | H |
| Le Gall | SAPS II | 0 | 0 | 0 | 1 | 1 | 0 | H | H |
| Lemeshow | MPM II models | 0 | U | 1 | 1 | 1 | 0 | H | H |
| Signorini | Signorini | 0 | 0 | 0 | 1 | 0 | 1 | H | H |
| Hukkelhoven | Hukkelhoven model | 0 | 0 | 0 | 0 | 0 | 0 | L | L |
| Maas | Rotterdam CT score | 0 | 0 | 0 | 0 | U | 0 | U | U |
| Perel | CRASH models | 0 | 0 | 0 | 0 | 0 | 0 | L | Ua |
| Steyerberg | IMPACT models | 0 | 0 | 0 | 0 | 0 | 0 | L | L |
| Jacobs | Nijmegen models | 0 | 0 | U | 0 | 1 | 1 | H | H |
| Yuan | Yuan models | 0 | 0 | 0 | 1 | 1 | 0 | H | H |
| A shorm form based on the PROBAST including 8/20 signaling questions consistent with Venema et al (2021). One point was assigned for each item that was incorrectly performed, resulting in total scores ranging from 0 to 6 points. Models with a total score of 0 were classified as ‘low RoB’(L) and models with a score ≥1 as ‘high ROB’ (H). When the total score was 0 but there was insufficient information provided to assess all items, the model was rated ‘unclear ROB’ (U).  ^a^ Unclear RoB based on the original PROBAST because the final model coefficients were not reported (Item 4.9) | | | | | | | | | |

| **Supplementary Table 7:** *Data extraction of validation studies of 18 prediction models for outcome following moderate and severe traumatic brain injury* | | | | | | | | | |
| --- | --- | --- | --- | --- | --- | --- | --- | --- | --- |
|  |  | **Source of data** | **Setting** | **Inclusion criteria** | **Outcome** | **Sample size** | **Model performance** | | **Relatedness** |
| **First author; Model** | **Study** | **Study design** | **Country; Stratum** |  | **Outcome;**  **Timing** | **Total number of patients; N events** | **Discrimination: AUC/C-statistic** | **Calibration:** |  |
| **Hukkelhoven model** | | | | | | | | | |
| Hukkelhoven;  Hukkelhoven model | DEV  Hukkelhoven, 2005 | Two multi-center clinical trials; Tirilazad | Europe, Israel, Australia, U.S.,  Canada | 15–65 years; severe (GCS 3–8) or moderate (GCS 9–12) closed TBI | Mortality,  Unfavorable outcome; 6mo | 2269;  Mortality: 500  Unfav: 862 | Mortality:  0.78 (0.76-0.81)  Unfav:  0.80 (0.78-0.82) | Hosmer-Lemeshow test: p-value  Mortality  0.06  Unfav  0.02 | NA |
|  | VAL  Hukkelhoven, 2005 | Multi-center observational cohort study:  European Brain Injury Consortium | Europe | Severe or moderate TBI | Mortality,  Unfavorable outcome; 6mo | 796;  Mortality: 244  Unfav: 388 | Mortality:  0.87 (0.84-0.89)  Unfav:  0.83 (0.80-0.86) | Hosmer-Lemeshow test: p-value  Mortality  0.42  Unfav  0.05 | Related |
|  | VAL  Hukkelhoven, 2005 | Multi-center observational cohort study:  Traumatic Coma Data Bank | North America | Non-penetrating severe TBI (GCS ≤8) | Mortality; 6mo | 746;  Mortality: 326 | Mortality:  0.89 (0.87-0.91) | Hosmer-Lemeshow test: p-value <.001 | Distantly related |
|  | VAL  Hukkelhoven, 2006 | Clinical trial:  Selfotel | Europe, Canada, Australia, Argentina | 16–65 years, severe (GCS 3–8) TBI, CT abnormalities, at least one reactive pupil, and non-penetrating head injury. | Mortality,  Unfavorable outcome; 6mo | 409;  Mortality: 94  Unfav: 177 | Mortality:  0.74 (0.68-0.80)  Unfav:  0.74 (0.69-0.79) | Hosmer-Lemeshow test: p-value  Mortality  0.49  Unfav  0.95 | Related |
|  | VAL  Hukkelhoven, 2006 | Multi-center observational cohort study:  EBIC | Europe | >16 years, Severe and moderate TBI | Mortality,  Unfavorable outcome; 6mo | 796;  Mortality: 244  Unfav: 388 | Mortality:  0.87 (0.84-0.89)  Unfav:  0.83 (0.80-0.86) | Hosmer-Lemeshow test: p-value  Mortality  0.42  Unfav  0.05 | Distantly related |
|  | VAL  Hukkelhoven, 2006 | Multi-center observational cohort study:  TCDB | US | >16 years, Non-penetrating TBI | Mortality; 6mo | 746;  Mortality: 326 | Mortality:  0.89 (0.86-0.91) | Hosmer-Lemeshow test: p-value <.01 | Distantly related |
|  | VAL  Harrison, 2015 | Multi-center observational cohort study | UK; Critical care unit | pre-sedation GCS <15 | Mortality,  Unfavorable outcome; 6mo | 2975;  Mortality: 764  Unfav: 1707 | Mortality:  0.78 (0.76-0.81)  Unfav:  0.71 (0.69-0.73) | Plot provided | Distantly related |
|  | VAL  Harrison, 2015 | Multi-center observational cohort study | UK; Critical care unit | “eligible for model” | Mortality, Unfavorable outcome; 6mo | 2975;  Mortality: 764  Unfav: 1707 | Mortality:  0.77 (0.75-0.80)  Unfav:  0.69 (0.66-0.72) | Plot provided | Moderately related |
| **Nijmegen models** | | | | | | | | | |
| Jacobs;  Nijmegen   - Clinical - Combination - CT | DEV  Jacobs 2012 | Multi-center observational cohort study: POCON | The Netherlands; Emergency department | >16 years; moderate (9-12) and severe (≤8) TBI | Mortality; Unfavorable outcome; Unfavorable outcome excluding death (GOSE 2–4); 6mo | 700;  Mortality: 251  Unfav: 328  Unfav excl. death: 77 | Mortality:  Clinical:  0.84  Combination:  0.85  CT:  0.90  Unfav:  Clinical:  0.82  Combination:  0.82  CT:  0.87  Unfav excl. death:  Clinical:  0.65  Combination:  0.71  CT:  0.74 | Hosmer-Lemeshow test: p-value  Mortality  0.49  0.27  0.34  Unfav  0.53  0.21  0.21  Unfav excl. death  0.85  0.07  0.04 | NA |
| - Clinical - Combination - CT | VAL  Jacobs, 2012 | Multi-center observational cohort study: POCON | The Netherlands | ≥16 years; TBI and ED admission GCS score ≤13. When intubated at injury scene, GCS score obtained before  Intubation ( ≤13) was used. | Mortality vs survival; Unfavorable vs favorable outcome; Unfavorable outcome excluding death vs favorable outcome;6mo | 333;  Mortality: not reported  Unfav: not reported  Unfav excl. death: not reported | Mortality  Clinical:  0.82 (0.78-0.87)  Combination:  0.79 (0.74-0.84)  CT:  0.86 (0.82-0.90)  Unfav  Clinical:  0.81  Combinations:  0.78  CT:  0.83  Unfav excl. death:  Clinical:  0.61  Combination:  0.71  CT:  0.69 | Hosmer-Lemeshow test: p-value  Mortality  0.60  0.26  0.53  Unfav | Distantly related |
| - Clinical | VAL  Majdan, 2014 | Multi-center observational cohort study | Austria | GCS ≤12 within 48 hours after the accident and/or AIS score of head >2 | Mortality, Unfavorable outcome;6mo | 778;  Mortality: 212  Unfav: 265 | Mortality:  Clinical:  0.86 (0.82-0.91)  Unfav:  Clinical:  0.82 (0.77-0.87) | Plot provided | Moderately related |
| **APACHE II** | | | | | | | | | |
| Knaus;  APACHE II | DEV  Knaus | Multi-center observational cohort study | U.S.; ICU | Patients admitted to ICU | Mortality; in hospital | 5030;  Mortality: 993 | Mortality:  0.86 | Not reported | NA |
|  | VAL  Raj, 2014 | Multi-center observational cohort study: FICC | Finland; ICU | >15 years, GCS 3–13 | Mortality; 6mo | 781;  Mortality: 262 | Mortality:  0.79 (0.75-0.82) | Hosmer-Lemeshow test: p-value  >0.05 | Distantly related |
|  | VAL  Kandil, 2020 | Multi-center observational cohort study | Egypt; ICU | Moderate to severe traumatic brain injury | Mortality; 6mo | 104;  Mortality: 25 | Mortality:  0.88 (0.81–0.94) | Not reported | Distantly related |
|  | VAL  Gürsoy, 2020 | Single-center observational cohort study | Turkey; ICU | Mild, moderate and severe traumatic brain injury; GCS 3-15 | Mortality; | 78;  Mortality: 27 | Mortality:  0.797 (SE 0.049) | Hosmer-Lemeshow test: p-value  >0.05 | Distantly related |
|  | VAL  Godino,  2020 | Multi center-observational cohort study | Uruguay; Neurocritical unit | GCS 3-15 (assumed) | Mortality; | 731;  Mortality: Not reported | Mortality:  0.79 | Not reported | Distantly related |
|  | VAL  Godino,  2020 | Multi center-observational cohort study | Uruguay; Neurocritical unit | GCS < 8 | Mortality; | Not reported;  Mortality: Not reported | Mortality:  0.79 | Not reported | Distantly related |
|  | VAL  Godino,  2020 | Multi center-observational cohort study | Uruguay; Neurocritical unit | Head trauma | Mortality; | 637;  Mortality: 220 | Mortality:  0.79 | Not reported | Distantly related |
|  | VAL  Godino,  2020 | Multi center-observational cohort study | Uruguay; Neurocritical unit | Subarachnoid hemorrhage | Mortality; | 236;  Mortality: 142 | Mortality:  0.73 | Not reported | Distantly related |
|  | VAL  Godino,  2020 | Multi center-observational cohort study | Uruguay; Neurocritical unit | Ischemic stroke | Mortality; | 398;  Mortality: 279 | Mortality:  0.68 | Not reported | Distantly related |
|  | VAL  Godino,  2020 | Multi center-observational cohort study | Uruguay; Neurocritical unit | Hemorrhagic stroke | Mortality; | 321;  Mortality: 211 | Mortality:  0.60 | Not reported | Distantly related |
|  | VAL  Godino,  2020 | Multi center-observational cohort study | Uruguay; Neurocritical unit | Anoxic Ischemic stroke | Mortality; | 352;  Mortality: 231 | Mortality:  0.85 | Not reported | Distantly related |
| **SAPS II** | | | | | | | | | |
| Le Gall;  SAPS II | DEV  Le Gall | Multi-center observational cohort study | Europe and North America; ICU | >18 years of age, admitted to ICU | Mortality; discharge | 8369;  Mortality: 1836 | Mortality:  0.86 (0.84-0.88) | Hosmer-Lemeshow test: p-value 0.10 | NA |
|  | VAL  Raj, 2014 | Multi-center observational cohort study: FICC | Finland; ICU | >15 years, GCS 3–13 | Mortality; 6mo | 781;  Mortality: 262 | Mortality:  0.80 (0.77-0.83) | Hosmer-Lemeshow test: p-value  >0.05 | Distantly related |
|  | VAL  Fischler, 2007 | Multi-center observational cohort study | Switzerland; ICU | Severe TBI: GCS <8 at the site of accident before being intubated or relevant head injury needing craniotomy | Mortality; discharge | 299;  Mortality: 24 | Mortality:  0.51 (0.37–0.70) | Not reported | Distantly related |
|  | VAL  Kandil,  2020 | Multi-center observational cohort study | Egypt; ICU | Moderate to severe traumatic brain injury | Mortality; 6mo | 104;  Mortality: 25 | Mortality:  0.87 (0.78–0.96) | Not reported | Distantly related |
|  | VAL  Godino,  2020 | Multi center-observational cohort study | Uruguay; Neurocritical unit | GCS 3-15 (assumed) | Mortality; | 538;  Mortality: Not reported | Mortality:  0.72 | Not reported | Distantly related |
|  | VAL  Godino,  2020 | Multi center-observational cohort study | Uruguay; Neurocritical unit | GCS < 8 | Mortality; | Not reported;  Mortality: Not reported | Mortality:  0.71 | Not reported | Distantly related |
|  | VAL  Godino,  2020 | Multi center-observational cohort study | Uruguay; Neurocritical unit | Head trauma | Mortality; | 637;  Mortality: 220 | Mortality:  0.75 | Not reported | Distantly related |
|  | VAL  Godino,  2020 | Multi center-observational cohort study | Uruguay; Neurocritical unit | Subarachnoid hemorrhage | Mortality; | 236;  Mortality: 142 | Mortality:  0.53 | Not reported | Distantly related |
|  | VAL  Godino,  2020 | Multi center-observational cohort study | Uruguay; Neurocritical unit | Ischemic stroke | Mortality; | 398;  Mortality: 279 | Mortality:  0.59 | Not reported | Distantly related |
|  | VAL  Godino,  2020 | Multi center-observational cohort study | Uruguay; Neurocritical unit | Hemorrhagic stroke | Mortality; | 321;  Mortality: 211 | Mortality:  0.66 | Not reported | Distantly related |
|  | VAL  Godino,  2020 | Multi center-observational cohort study | Uruguay; Neurocritical unit | Anoxic Ischemic stroke | Mortality; | 352;  Mortality: 231 | Mortality:  0.65 | Not reported | Distantly related |
|  | VAL  Godino,  2020 | Multi center-observational cohort study | Uruguay; Neurocritical unit | GCS 3-15 (assumed) | Mortality; | 731;  Mortality: Not reported | Mortality:  0.72 | Not reported | Distantly related |
| **MPM II** | | | | | | | | | |
| Lemeshow;  MPM II   - MPM0 - MPM24 | DEV  Lemeshow, | Multi-center observational cohort study; ICU | U.S. | >18 years, admitted to ICU | Mortality; in hospital | 6514;  Mortality: 2261 | Mortality:  MPM0  0.82  MPM24  0.84 | Hosmer-Lemeshow test: p-value  MPM0  0.33  MPM24  0.23 | NA |
|  | VAL  Fischler, 2007 | Multi-center observational cohort study; ICU | Switzerland; ICU | Severe TBI: GCS <8 at the site of accident before being intubated or relevant head injury needing craniotomy | Mortality; discharge | 299;  Mortality: 24 | Mortality:  MPM0  0.47 (0.34–0.66)  MPM24  0.57 (0.41–0.79) | Not reported | Distantly related |
| **Rotterdam CT score** | | | | | | | | | |
| Maas;  Rotterdam CT score | DEV  Maas | Two multi-center clinical trials | U.S. | 15-65 years; severe (GCS 3–8) or moderate (GCS 9–12) closed TBI. CT characteristics: the international study excluded moderate TBI patients with a normal CT scan, whereas the North American study excluded such patients only when the blood alcohol level exceeded 0.2 g/dl. | Mortality; 6mo | 2269;  Mortality: 491 | Mortality:  0.75 | Not reported | NA |
|  | VAL  Charry, 2017 | Single-center observational cohort study | Colombia | >17 years, Severe TBI (≤8) | Mortality; 6mo | 127;  Mortality: 37 | Mortality:  0.83 (0.75–0.90) | Not reported | Distantly related |
|  | VAL  Majdan,  2017 | Multi-center observational cohort studies | Austria | Moderate and severe TBI | Mortality, Unfavorable outcome; 6mo | 866;  Mortality: 359  Unfav: 427 | Mortality;  0.70  Unfav;  0.72 | Not reported | Related |
|  | VAL  Charry, 2019 | Single-center observational cohort study | Colombia | >18 years; Mild, moderate and severe TBI | Mortality; 6mo | 309;  Mortality: 39 | Mortality;  0.88 (0.81-0.94) | Not reported | Distantly related |
|  | VAL  Elkbuli, 2021 | Single-center observational cohort study | US; ICU | ≥18 years; Severe TBI; GCS 3-5 | Mortality; Not reported | 106;  Mortality: 43 | Not reported | Not reported | Distantly related |
| **CRASH** | | | | | | | | | |
| Perel;  CRASH   - Basic low income country - Basic high income country - CT low income country - CT high income country | DEV  Perel, 2008 | Multi-center clinical trial | U.S. | ≥16 years; GCS ≤14; Within eight hours of injury | Mortality, Unfavorable outcome; 14 days and 6mo | 10008;  Mortality: 1948  Unfav: 3556 | Mortality;  Basic:  0.84  Basic:  0.86  CT:  0.84  CT:  0.88  Unfav;  Basic:  0.84  Basic:  0.81  CT:  0.84  CT:  0.83 | Hosmer-Lemeshow test: p-value  Mortality  Basic: 0.40  Basic:0.27  CT: 0.04  CT: 0.60  Unfav  Basic: 0.20  Basic:0.70  CT: 0.03  CT: 0.63 | NA |
| - Basic high income - CT high income | VAL  Perel, 2008 | Eight randomized controlled trials, three observational cohort studies: IMPACT | International | ≥ 14 years; moderate and severe TBI (GCS ≤ 12) | Unfavorable outcome; 6mo | 10008; Not reported | Unfav;  Basic:  0.77  CT:  0.77 | Plot provided | Distantly related |
| - CT | VAL  Honeybul, 2009 | Multi-center observational cohort | Australia | Severe head injury; decompressive craniectomy | Mortality, Unfavorable outcome;6mo | 41;  Mortality: 7  Unfav: 18 | Mortality;  CT:  0.76 (0.54-0.98)  Unfav;  CT:  0.94 (0.85-1.00) | Hosmer-Lemeshow test: p-value  Mortality:  Not reported  Unfav:  0.05 | Distantly related |
| - Basic | VAL  Güiza, 2013 | Multi-center observational cohort: Brain-IT | Europe; Neuro ICU | TBI | Poor neurological outcome (GOS 1-2); 6mo | 160;  Mortality: 29 | Poor neuro;  Basic:  0.72 (0.68–0.74) | Hosmer-Lemeshow test: p-value  0.43 | Distantly related |
| - Basic | VAL  Wong, 2013 | Single-center observational cohort | China | Mild, moderate, and severe TBI (loss of consciousness or change in consciousness level with head and neck Abbreviated Injury Severity score ≥2) | Mortality, Unfavorable outcome; 14 days and 6mo | 310;  Mortality: 79  Unfav: 133 | Mortality;  Basic:  0.89 (0.85–0.92)  Unfav;  Basic:  0.89 (0.85–0.92) | Mortality  Intercept:-0.33  Slope:1.9  Unfav  Intercept:-0.56  Slope:2.51 | Moderately related |
| - Basic - CT | VAL  Han, 2014 | Single-center observational cohort | Singapore; ICU | severe TBI (GCS ≤ 8) | Mortality, Unfavorable outcome; 14 days and 6mo | 300;  Mortality: 143  Unfav: 213 | Mortality;  Basic:  0.80 (0.75–0.85)  CT:  0.83 (0.75–0.86)  Unfav;  0.86 (0.81–0.90)  CT:  0.89 (0.84–0.93) | Hosmer-Lemeshow test: p-value  Mortality  Basic:  > 0.05  Intercept:0.51  Slope:0.95  CT:  > 0.05  Intercept:0.03  Slope:0.85  Unfav  Basic:  > 0.05  Intercept:-1.27  Slope:0.84  CT:  > 0.05  Intercept:0.21  Slope:0.87 | Distantly related |
| - CT | VAL  Honeybul, 2014 | Multi-center observational cohort | Australia; ICU and ward | Severe TBI; requiring decompressive craniectomy | Mortality, Unfavorable outcome; 18mo | 270;  Mortality: 45  Unfav: 153 | Mortality;  CT:  0.79 (0.72– 0.86)  Unfav;  CT:  0.85 (0.80–0.90) | Hosmer-Lemeshow test: p-value  Mortality  0.57  Intercept:-1.27  Slope:0.84  Unfav  0.03  Intercept:-1.11  Slope:1.66 | Distantly related |
| - Basic | VAL  Majdan, 2014 | Multi-center observational cohort | Austria | GCS ≤12 within 48 hours after the accident and/or AIS score of head >2 | Mortality, Unfavorable outcome; 6mo | 778;  Mortality: 212  Unfav: 265 | Mortality:  Basic:  0.82 (0.77-0.87)  Unfav;  Basic:  0.80 (0.75-0.84) | Plot provided  Mortality  Intercept:0.21  Slope:0.87  Unfav  Intercept:-0.50  Slope:0.87 | Distantly related |
| - CT | VAL  Bonds, 2015 | Single-center observational cohort | U.S. | ≥14 years, moderate to severe TBI (admission Glasgow Coma Score (GCS) ≤12, and a head abbreviated injury scale (AIS) score ≥3) | Mortality, Unfavorable outcome; 14 days and 6mo | 86;  Mortality: 16  Unfav: 65 | Mortality:  CT:  0.89  Unfav;  CT:  0.86 | Hosmer-Lemeshow test: p-value  Mortality  <0.0001  Unfav  <0.0001 | Distantly related |
| - Basic - CT | VAL  Roe, 2015 | Multi-center observational cohort | Norway | ≥65; GCS=3-8 | Mortality, Unfavorable outcome excl. deaths; 14 days and 12mo | 97;  Mortality: 48  Unfav excl. deaths: 70 | Not reported | Not reported | Distantly related |
| - Basic - CT | VAL  Harrison, 2015 | Multi-center observational cohort | UK; Critical care unit | pre-sedation GCS <15 | Unfavorable outcome; 6mo | 2975;  Unfav: 2422 | Unfav;  Basic:  0.699 (0.677-0.721)  CT:  0.708 (0.686-0.729) | Plot provided | Distantly related |
| - Basic - CT | VAL  Harrison, 2015 | Multi-center observational cohort | UK; Critical care unit | ‘’Eligible for model’’ | Unfavorable outcome; 6mo | 2975;  Unfav: 2422 | Unfav;  Basic:  0.70 (0.68-0.72)  CT:  0.71 (0.69-0.73) | Plot provided | Moderately related |
| - Basic refitted - CT refitted | VAL  Castano-Leon, 2016 | Single-center observational cohort | Spain; ICU | >14 years; blunt severe or moderate TBI GCS <13 | Mortality, Unfavorable outcome; 14 days and 6mo | 1301;  Mortality: 549  Unfav: 931 | Mortality:  Basic:  0.80 (0.81-0.85) CT:  0.83 (0.81-0.86)  Unfav:  Basic:  0.78 (0.75-0.80)  CT:  0.79 (0.77-0.82) | Mortality  Basic  Intercept:-0.94  Slope:0.85  CT  Intercept:-1.1  Slope:0.95  Unfavorable  Basic  Intercept:-0.33  Slope:0.59  CT  Intercept:-0.46  Slope:0.61 | Distantly related |
| - Basic - CT | VAL  Hashemi, 2016 | Multi-center observational cohort | Iran; ED | GCS≤ 14 | Mortality, Unfavorable outcome; 14 days and 6mo | 323;  Mortality: 64  Unfav: 81 | Mortality:  Basic:  0.92 (0.89–0.96)  CT:  0.93 (0.91–0.97)  Unfav:  Basic:  0.92 (0.90–0.95)  CT:  0.93 (0.91–0.96) | Hosmer-Lemeshow test: p-value  Mortality:  Basic  0.07  CT  0.07  Unfav:  Basic  0.15 CT  0.08 | Moderately related |
| - CT | VAL  Honeybul, 2016 | Multi-center observational cohort | Australia | Severe TBI; decompressive craniectomy | Mortality, Unfavorable outcome; 18mo | 319;  Mortality: 53  Unfav: 199 | Mortality:  CT:  0.79 (0.72–0.86)  Unfav:  CT:  0.86 (0.81–0.90) | Hosmer-Lemeshow test: p-value  Mortality:  CT  0.86  Unfav:  CT  0.03  Mortality:  CT  Intercept:-3.2*  Slope: 1.05*  Unfav: CT  Intercept:-5.7*  Slope: 1.09* | Distantly related |
| - CT | VAL  Charry, 2017^b^ | Single-center observational cohort | Colombia | Severe TBI; Decompressive craniectomy | Mortality, Unfavorable outcome; 6mo | 127;  Mortality: 37  Unfav: 47 | Mortality:  CT:  0.71 (0.59-0.82)  Unfav:  CT:  0.71 (0.59-0.82) | Not reported | Distantly related |
| - Basic | VAL  Faried, 2018 | Single-center observational cohort | Indonesia; Neurosurgery unit | Diagnosis of TBI; Exclusion criteria: GCS score of 15, pediatric patients, >8-hour time interval between trauma onset and hospital arrival, patients not admitted or voluntarily discharged, and unavailability of head computed tomography scan data | Mortality; 14 days | 229;  Mortality: 60 | Not reported | Not reported | Moderately related |
| - CT | VAL  Sadaka, 2018 | Single-center observational cohort | U.S. | GCS=3 | Mortality, Unfavorable outcome; 14 days and 6mo | 62;  Mortality: 50  Unfav: Not reported | Mortality:  CT:  0.94 (0.89-0.99)  Unfav:  CT:  1.00 (1.00-1.00) | Not reported | Distantly related |
| - CT | VAL  Sadaka, 2018 | Single-center observational cohort | U.S. | GCS≤ 14 | Mortality, Unfavorable outcome; 14 days and 6mo | 416:  Mortality: 77  Unfav: Not reported | Mortality:  CT:  0.91 (0.90-0.92)  Unfav:  CT:  0.85 (0.83-0.87) | Not reported | Related |
| - Basic | VAL  Maeda, 2019 | Multi-center observational cohort: JNTDB | Japan | GCS score ≤8 | Unfavorable outcome; 6mo | 511:  Unfav: 293 | Unfav:  Basic:  0.86 (0.82–0.90) | Not reported | Distantly related |
| - CT | VAL  Charry, 2019 | Single-center observational cohort | Colombia | >18 years; Mild, moderate and severe TBI | Mortality, Unfavorable outcome; 6mo | 309;  Mortality: 39  Unfav: 53 | Mortality:  CT:  0.88 (0.83-0.93)  Unfav:  CT:  0.83 (0.79-0.88) | Not reported | Moderately related |
| - Basic - CT | VAL  Wongchareon, 2020 | Multi-center trial and observational cohort | South America; ICU | severe TBI | Mortality, Unfavorable outcome; 14days and 6mo | 466;  Mortality: 122  Unfav: 264 | Mortality:  Basic  0.640 (0.597-0.682)  CT  0.661 (0.620-0.703)  Unfav:  Basic  0.718 (0.685-0.751)  CT  0.731 (0.699-0.763) | Hosmer-Lemeshow test: p-value  Mortality  Basic  0.05  Intercept:0.10  Slope:0.76  CT  0.001  Intercept: 0.07 Slope:1.05  Unfav  Basic  0.00  Intercept:0.17  Slope:0.88  CT  0.04  Intercept:0.17  Slope:0.98 | Distantly related |
| - Basic - CT | VAL  Mikolic, 2021 | Multi-center observational cohort | Europe, Israel; ED, ward and ICU | Mild TBI (GCS 13-15) | Unfavorable outcome; 6mo | 2269  Unfav Basic: 259  2064  Unfav CT: 233 | Unfav:  Basic  0.78 (0.74-0.81)  CT  0.79 (0.75-0.82) | Basic  Mbc: 0.79  Intercept:-0.82  Slope:0.96  CT  Mbc: 0.80  Intercept:-1.38  Slope:0.90 | Distantly related |
| - Basic - CT | VAL  Mikolic, 2021 | Multi-center observational cohort study | Europe, Israel; ED, ward and ICU | Mild TBI (GCS 13-14) | Unfavorable outcome; 6mo | 558;  Unfav Basic: 111  492;  Unfav CT: 93 | Unfav:  Basic  0.76 (0.76-0.87)  CT  0.78 (0.73-0.84) | Basic  Mbc: 0.79  Intercept:-0.26  Slope:0.95  CT  Mbc: 0.82  Intercept:-1.13  Slope:0.82 | Distantly related |
| - Basic | VAL  Dullaert, 2020 | Single-center observational cohort study | Belgium;  ED | Adult patients; Mild, moderate and severe TBI; GCS 3-15 | Mortality; 14 days;  Unfavorable outcome; 6mo | 417;  Mortality: 83  307;  Unfav: 233 | Mortality:  Basic  0.92  Unfav:  Basic  0.90 | Not reported | Distantly related |
| - Basic | VAL  Camarano, 2021 | Multi-center observational cohort study | U.S., Canada; | GCS 3-12 | In-hospital mortality or discharge to hospice | 26.228;  Mortality: 10.919 | Mortality:  Basic  0.858 (0.854-0.863) | Not reported | Distantly related |
| - Basic | VAL  Camarano, 2021 | Multi-center observational cohort study | U.S., Canada; | GCS 3-12 | In-hospital mortality | 26.228;  Mortality: 10.167 | Mortality:  Basic  0.847 (0.842-0.851) | Not reported | Distantly related |
| - Basic | VAL  Camarano, 2021 | Multi-center observational cohort study | U.S., Canada; | GCS 3-14 | In-hospital mortality or discharge to hospice | Not reported | Mortality:  Basic  0.872 (0.869-0.876) | Not reported | Distantly related |
| - Basic | VAL  Elahi, 2021 | Single-center observational cohort study | Tanzania; | Mild, moderate and severe TBI; GCS 3-15 | Unfavorable outcome (GOS 1-3) | 2972;  Unfav: 327 | Unfav:  Basic  0.876 (0.852-0.90) | Hosmer-Lemeshow test: p-value  <.001 | Distantly related |
| - Basic - CT | VAL  Dijkland,  2021 | Multi-center observational cohort study | Europe, Israel; ED, ward and ICU | Age ≥ 16;  GCS ≤ 14 | Mortality; 14 days;  Unfavorable outcome; 6mo | Basic  1754;  Mortality:  Unfav:  CT  1542;  Mortality:  Unfav: | Mortality:  Basic  0.86 (0.83-0.88)  CT  0.88 (0.86-0.90)  Unfav:  Basic  0.82 (0.80-0.84)  CT  0.84 (0.82-0.86)  Mbc  Mortality:  Basic  0.86 (0.84-0.88)  CT  0.91 (0.87-0.91)  Unfav:  Basic  0.83 (0.81-0.85)  CT  0.86 (0.84-0.88) | Mortality  Basic  Intercept:-0.01  Slope:0.95  CT  Intercept:-2.02  Slope:0.75  Unfav  Basic  Intercept:-0.02  Slope:0.97  CT  Intercept:-0.93  Slope:0.85 | Moderately related |
| **Signorini model** | | | | | | | | | |
| Signorini;  Signorini model | DEV  Signorini, 1999 | Single-center cohort study | UK | ≥14 years; GCS <= 12, or GCS 13-15 with ISS >= 16 or more | Survival; 12mo | 365;  Survival: 278 | Survival:  0.90 | Hosmer-Lemeshow test: p-value  0.13 | NA |
|  | VAL  Signorini, 2014 | Not reported | Not reported | ≥14 years; GCS <= 12, or GCS 13-15 with ISS >= 16 or more | Survival; 12mo | 520;  Survival: Not reported | Survival:  0. 84 | Hosmer-Lemeshow test: p-value  > 0.0001 | Cannot judge |
|  | VAL  Hukkelhoven, 2006 | Two multi-center clinical trials;  Tirilazad | Europe, Israel, Australia, U.S.,  Canada | 15–65 years; severe (GCS 3–8) or moderate (GCS 9–12) closed TBI | Mortality,  Unfavorable outcome; 6mo | 2269;  Mortality: 500  Unfav: 862 | Mortality:  0.71 (0.69,0.74) | Hosmer-Lemeshow test: p-value  Mortality:  < 0.01 | Distantly related |
|  | VAL  Hukkelhoven, 2006 | Clinical trial: Selfotel | Europe, Canada, Australia, Argentina | 16–65 years, severe (GCS 3–8) TBI, presence of abnormalities on the CT scan, at least one reactive pupil, and non-penetrating head injury. | Mortality,  Unfavorable outcome; 6mo | 409;  Mortality: 94  Unfav: 177 | Mortality:  0.61 (0.54, 0.68) | Hosmer-Lemeshow test: p-value  Mortality:  < 0.01 | Distantly related |
|  | VAL  Hukkelhoven, 2006 | Multi-center observational cohort study:  EBIC | Europe | >16 years, Severe and moderate TBI | Mortality,  Unfavorable outcome; 6mo | 796;  Mortality: 244  Unfav: 388 | Mortality:  0.85 (0.82, 0.88) | Hosmer-Lemeshow test: p-value  Mortality:  < 0.01 | Distantly related |
|  | VAL  Hukkelhoven, 2006 | Multi-center observational cohort study:  TCDB | U.S. | >16 years, Non-penetrating TBI | Mortality; 6mo | 746;  Mortality: 326 | Mortality:  0.81 (0.78, 0.84) | Hosmer-Lemeshow test: p-value  Mortality:  < 0.01 | Distantly related |
| **IMPACT models** | | | | | | | | | |
| Steyerberg;  IMPACT   - Core - Extended - Lab | DEV  Steyerberg  2008 | Randomized controlled trials: TirInt and TirUS and observational cohort studies | International U.S.  UK  Europe | ≥ 14 years; moderate and severe TBI (GCS ≤ 12) | Mortality, Unfavorable outcome; 6mo | 8509;  Mortality: 2396  Unfav: 5748 | Mortality:  Core:  0.70 TirInt  0.74 TirUS  0.66 Saphir  0.76 Pegsod  0.72 HIT-II  0.81 TCDB  0.81 cohort UK  0.84 EBIC  Extended:  0.78 TirInt  0.77 TirUS  0.71 Saphir  0.77 HIT-II  0.81 TCDB  0.83 cohort UK  0.87 EBIC  Lab:  0.80 TirInt  0.79 TirUS  0.72 Saphir  Unfav:  Core:  0.70 TirInt  0.74 TirUS  0.66 Saphir  0.76 Pegsod  0.72 HIT-II  0.81 TCDB  0.81 cohort UK  0.84 EBIC  Extended:  0.78 TirINT  0.77 TirUS  0.71 Saphir  0.77 HIT-II  0.81 TCDB  0.83 cohort UK  0.87 EBIC  Lab:  0.80 TirINT  0.79 TirUS  0.72 Saphir | Not reported | NA |
| - Core - Ext | VAL  Steyerberg, 2008 | Multi-center clinical trial: CRASH | U.S. | All with GCS ≤12 | Mortality, Unfavorable outcome; 6mo | 5309;  Mortality: Not reported  Unfav: Not reported | Mortality:  Core:  0.78  Ext:  0.80  Unfav:  Core:  0.78  Ext:  0.80 | Not reported | Related |
| - Core - Ext | VAL  Steyerberg, 2008 | Multi-center clinical trial: CRASH | U.S. | Placebo patients | Mortality, Unfavorable outcome; 6mo | 2616;  Mortality: Not reported  Unfav: Not reported | Mortality:  Core:  0.78  Ext:  0.81  Unfav:  Core:  0.78 Ext:  0.79 | Not reported | Related |
| - Core - Ext | VAL  Steyerberg, 2008 | Multi-center clinical trial: CRASH | U.S\| | High income countries | Mortality, Unfavorable outcome; 6mo | 1351;  Mortality: Not reported  Unfav: Not reported | Mortality:  Core:  0.80  Ext:  0.83  Unfav:  0.77  0.80 | Not reported | Related |
| - Core | VAL  Yeoman, 2010 | Single-center observational cohort | UK | Moderate and severe TBI GCS ≤ 12 | Mortality, Unfavorable outcome; 12mo | 1061;  Mortality: 390  Unfav: 555 | Mortality:  Core:  0.83  Unfav:  Core:  0.82 | Plot provided | Distantly related |
| - Core - Ext - Lab | VAL  Olivecrona, 2012 | Consecutive double-blinded randomized study | Sweden | verified blunt head trauma, GCS at intubation and sedation ≤8 and age 15–70 years | Mortality, Unfavorable outcome; 3mo | 48;  Mortality: 7  Unfav: 22 | Mortality:  Core:  0.74  Ext:  0.75  Lab:  0.78  Unfav:  Core:  0.73  Ext:  0.72  Lab:  0.81 | Not reported | Distantly related |
| - Core - Ext - Lab | VAL  Panczykowski, 2012 | Single-center observational cohort | U.S. | Severe TBI (GCS ≤ 8) | Mortality, Unfavorable outcome; 6mo | 587;  Mortality: 241  Unfav: 431 | Mortality:  Core:  0.78  Ext:  0.83  Lab:  0.83  Unfav:  Core:  0.76  Ext:  0.78  Lab:  0.76 | Hosmer-Lemeshow test: p-value  Mortality  Core  0.70  Ext  0.89  Lab  0.80  Unfav  Core  0.39  Ext  0.17  Lab  0.78 | Distantly related |
| - Core - Ext | VAL  Roozenbeek, 2012 | Multi-center observational cohort | U.S. | Severe TBI (GCS ≤ 8) | Mortality; 14 days | 2513;  Mortality: 578 | Mortality:  Core:  0.79 (0.77-0.81)  Ext:  0.83 (0.81-0.85) | Plot provided  Mortality  Intercept:-0.60  Slope:1.06  Ext  Intercept:0.03  Slope:1.12 | Distantly related |
| - Core | VAL  Güiza, 2013 | Multi-center observational cohort | Europe | TBI | Poor neurological outcome (GOS 1-2); 6mo | 160;  Poor neurological outcome: 29 | Poor neurological outcome:  Core:  0.72 (0.69–0.75) | Hosmer-Lemeshow test: p-value  0.51  Intercept:-.001  Slope:0.81 | Distantly related |
| - Core | VAL  Raj, 2013 | Single-center observational cohort | Finland | 14- 99 years; admission GCS 3-12; exclusion of penetrating head injuries. | Mortality, Unfavorable outcome excl. deaths; 6mo | 342;  Mortality: 110  Unfav excl. deaths: 78 | Mortality:  Core:  0.85 (0.81-0.89)  Unfav:  Core:  0.81 (0.76-0.86) | Hosmer-Lemeshow test: p-value  Mortality  0.35  Unfav  0.27 | Moderately related |
| - Core | VAL  Wong, 2013 | Single-center observational cohort | China | TBI (loss of consciousness or change in consciousness level with a head and neck Abbreviated Injury Severity score ≥2) | Mortality, Unfavorable outcome; 6mo | 178;  Mortality: 64  Unfav: 110 | Mortality:  Core:  0.80 (0.73–0.86)  Unfav:  Core:  0.81 (0.75–0.87) | Plot provided  Mortality  Intercept:-0.07  Slope:2.29  Unfav  Intercept:0.26  Slope:2.08 | Distantly related |
| - Core - Ext - Lab | VAL  Lingsma, 2013 | Multi-center observational cohort | The Netherlands | Severe TBI | Mortality, Unfavorable outcome; 6mo | 415;  Mortality: 169  Unfav: 311 | Mortality:  Core:  0.85 (0.81-0.88)  Ext:  0.88 (0.85-0.91)  Lab:  0.90 (0.87-0.92)  Unfav:  Core:  0.82 (0.79-0.86)  Ext:  0.85 (0.82-0.89)  Lab:  0.87 (0.83-0.90) | Plot provided  Mortality  Core  Intercept:0.08 Slope:1.46  Ext  Intercept:0.12 Slope:1.24  Lab  Intercept:0.72 Slope:1.57  Unfav  Core  Intercept:-0.37 Slope:1.24 | Distantly related |
| - Core - Ext - Lab | VAL  Han, 2014 | Single-center observational cohort | Singapore | Severe TBI (GCS ≤ 8) | Mortality, Unfavorable outcome; 6mo | 300;  Mortality: 164  Unfav: 213 | Mortality:  Core:  0.80 (0.75–0.85)  Ext:  0.81 (0.76–0.86)  Lab:  0.80 (0.75–0.86)  Unfav:  Core:  0.84 (0.80–0.89)  Ext:  0.88 (0.83–0.92)  Lab:  0.87 (0.82–0.92) | Hosmer-Lemeshow test: p-value  Mortality Core  >.05  Intercept:0.81 Slope:1.14  Ext  >.05  Intercept:0.59 Slope:1.15  Lab  >.05  Intercept:0.81 Slope:1.17  Unfav  Core  >.05  Intercept:1.07 Slope:1.37  Ext  >.05  Intercept:0.88 Slope:1.58  Lab  >.05  Intercept:0.94 Slope:1.46 | Distantly related |
| - Core - Ext | VAL  Majdan, 2014 | Multi-center observational cohort | Austria | GCS ≤12 within 48 hours after the accident and/or AIS score of head >2 | Mortality, Unfavorable outcome; 6mo | 778;  Mortality: 212  Unfav: 265 | Mortality: Core:  0.84 (0.8-0.87)  Ext:  0.85 (0.81-0.89)  Unfav:  Core:  0.80 (0.76-0.84)  Ext:  0.81 (0.77-0.86) | Not reported | Moderately related |
| - Core - Ext - Lab | VAL  Raj, 2014 | Single-center observational cohort | Finland: ICU | Moderate and severe TBI (GCS 3-12) or complicated mild TBI (GCS 13–15) | Mortality, Unfavorable outcome; 6mo | 842;  Mortality: 206  Unfav: 394 | Mortality:  Core:  0.80 (0.77–0.83)  Ext:  0.80 (0.77–0.83)  Lab:  0.81 (0.78–0.84)  Unfav:  Core:  0.78 (0.75–0.81)  Ext:  0.79 (0.76–0.82)  Lab:  0.79 (0.76–0.82) | Hosmer-Lemeshow test: p-value  Mortality  Core  <.001  Ext  <.001  Lab  0.054  Unfav  Core  <.001  Ext  .006  Lab  .078 | Distantly related |
| - Ext | VAL  Bonds, 2015 | Single-center observational cohort | U.S. | ≥14 years; moderate, severe TBI (admission Glasgow Coma Score (GCS) ≤12, and a head abbreviated injury scale (AIS) score ≥3) | Mortality, Unfavorable outcome; 14 days and 6mo | 86;  Mortality: 16  Unfav: 65 | Mortality:  Ext:  0.88  Unfav:  Ext:  0.87 | Hosmer-Lemeshow test: p-value  Mortality  Ext  <.0001  Unfav  Ext  <.0001 | Related |
| - Core - Ext - Lab | VAL  Harrison, 2015 | Multi-center observational cohort | UK | pre-sedation GCS <15 | Mortality, Unfavorable outcome; 6mo | 2975;  Mortality: 764  Unfav: 1707 | Mortality:  Core:  0.75 (0.73-0.77)  Ext:  0.78 (0.76-0.80)  Lab:  0.78 (0.76-0.80)  Unfav:  Core:  0.69 (0.67-0.72)  Ext:  0.71 (0.69-0.73)  Lab:  0.71 (0.69-0.74) | Plot provided | Distantly related |
| - Core - Ext - Lab | VAL  Harrison, 2015 | Multi-center observational cohort | UK | ‘Eligble for model’: GCS ≤ 12 | Mortality, Unfavorable outcome; 6mo | 2528;  Mortality: 685  Unfav: 1323 | Mortality:  Core:  0.75 (0.73-0.77)  Ext:  0.78 (0.76-0.80)  Lab:  0.78 (0.76-0.80)  Unfav:  Core:  0.69 (0.67-0.71)  Ext:  0.71 (0.68-0.73)  Lab:  0.71 (0.69-0.74) | Plot provided | Related |
| - Core - Ext | VAL  Castano-Leon, 2016 | Single-center observational cohort | Spain | >14 years; blunt severe or moderate TBI GCS <13 | Mortality, Unfavorable outcome; 14 days and 6mo | 1301;  Mortality: 549  Unfav: 931 | Mortality:  Core:  0.83 (0.81-0.85)  Ext:  0.87 (0.85-0.89)  Unfav:  Core:  0.80 (0.77-0.82)  Ext:  0.83 (0.80-0.85) | Mortality  Core  Intercept:0.93  Slope:1.5  Ext  Intercept:0.60  Slope:1.5  Mortality  Unfav  Intercept:0.77  Slope:1.17  Ext  Intercept:0.53  Slope:1.15 | Related |
| - Core - Lab | VAL  Staples, 2016 | Multi-center observational cohort | U.S. | 18-64 years; GCS ≤ 12 | Mortality; 6mo  Unfav: 3mo | 613;  Mortality: Not reported  Unfav: Not reported | Mortality:  Core:  0.81 (0.77–0.84) Lab:  0.83 (0.80–0.86) Unfav:  Lab:  0.80 (0.77–0.84) | Hosmer-Lemeshow test: p-value  Core  0.01  Lab  0.04 | Distantly related |
| - Core - Lab | VAL  Staples, 2016 | Multi-center observational cohort | U.S. | ≥65 years;  GCS ≤ 12 | Mortality; 6mo  Unfav: 3mo | 202;  Mortality: Not reported  Unfav: Not reported | Mortality:  Core:  0.75 (0.66–0.84)  Lab:  0.77 (0.68–0.86)  Unfav:  Lab:  0.84 (0.77–0.91) | Hosmer-Lemeshow test: p-value  Core  <.0001  Lab  <.0001 | Distantly related |
| - Core - Ext - Lab | VAL  Honeybul, 2016 | Multi-center observational cohort | Australia | Severe TBI; Decompressive craniectomy | Mortality, Unfavorable outcome; 18mo | 319;  Mortality: 53  Unfav: 119 | Mortality:  Core:  0.73 (0.65–0.81)  Ext:  0.76 (0.69–0.83)  Lab:  0.77 (0.71–0.84)  Unfav:  Core:  0.81 (0.76–0.86)  Ext:  0.85 (0.80–0.89)  Lab:  0.85 (0.80–0.89) | Hosmer-Lemeshow test: p-value  Mortality  Core  0.31  Intercept:-2.9*  Slope:1.05*  Ext  0.21  Intercept:-3.3*  Slope:1.05*  Lab  0.07  Intercept:-3.2*  Slope:1.05*  Unfav  Core  0.45  Intercept:-3.0*  Slope:1.07*  Ext  0.17  Intercept:-4.2*  Slope:1.08*  Lab  0.03  Intercept:-4.1*  Slope:1.08* | Distantly related |
| - Core - Ext - Lab | VAL  Sun, 2016 | Multi-center randomized controlled trial | North America, Europe, Asia,  South America | Severe TBI | Mortality, Unfavorable outcome; 6mo | 1124;  Mortality: 203  Unfav: 773 | Mortality:  Core:  0.68  Ext:  0.69  Lab:  0.69  Unfav:  Core:  0.68  Ext:  0.71  Lab:  0.71 | Plot provided  Mortality  Core  Intercept:-0.70  Slope:0.81  Ext  Intercept:-0.66  Slope:0.76  Lab  Intercept:-0.58  Slope:0.76  Unfav  Core  Intercept:0.23  Slope:0.72  Ext  Intercept:0.34  Slope:0.72  Lab  Intercept:0.42  Slope:0.71 | Moderately related |
| - Lab | VAL  Charry, 2017 | Single-center observational cohort | Colombia | Severe TBI; Decompressive craniectomy | Unfavorable outcome; 6mo | 127;  Unfav: 47 | Unfav:  Lab:  0.67 (0.58-0.76) | Not reported | Distantly related |
| - Ext | VAL  Majdan, 2017 | Multi-center observational cohort studies | Austria | Moderate and severe TBI | Mortality, Unfavorable outcome; 6mo | 866;  Mortality: 359  Unfav: 427 | Mortality:  Ext:  0.86  Unfav: Ext:  0.84 | Not reported | Moderately related |
| - Core - Ext - Lab | VAL  Wan, 2017 | Single-center observational cohort | China | Geriatric patients ≥65; Severe TBI | Mortality, Unfavorable outcome; 6mo | 137;  Mortality: 75  Unfav: 97 | Mortality:  Core:  0.76  Ext:  0.76  Lab:  0.73  Unfav:  Core:  0.80  Ext:  0.79  Lab:  0.77 | Hosmer-Lemeshow test: p-value  Mortality  Core  0.28  Ext  0.25  Lab  0.58  Unfav  Core  0.35  Ext  0.23  Lab  0.55 | Distantly related |
| - Core - Ext - Lab | VAL  Egea-Guerrero, 2018 | Multi-center observational cohort | Spain | GCS ≤ 12 | Mortality, Unfavorable outcome; 6mo | 290;  Mortality: 47  Unfav: 74 | Mortality:  Core:  0.84 (0.78-0.90)  Ext:  0.88 (0.83-0.93)  Lab:  0.90 (0.86-0.94)  Unfav:  Core:  0.84 (0.79-0.89)  Ext:  0.84 (0.79-0.89)  Lab:  0.83 (0.77-0.88) | Hosmer-Lemeshow test: p-value  Mortality  Core  0.87  Ext  0.10  Lab  0.80  Unfav  Core  0.15  Ext  0.42  Lab  0.28 | Moderately related |
| - Ext | VAL  Ho, 2018 | Multi-center observational cohort | Australia | Severe TBI; Decompressive craniectomy | Unfavorable outcome; 18mo | 56;  Unfav: 32 | Unfav:  Ext:  0.918 (0.809-0.976) | Not reported | Distantly related |
| - Lab | VAL  Charry, 2019 | Single-center observational cohort | Colombia | Older than 18 years; Mild, moderate and severe TBI | Mortality, Unfavorable outcome; 6mo | 309;  Mortality: 39  Unfav: 53 | Mortality:  Lab:  0.90 (0.86-0.94)  Unfav:  Lab:  0.86 (0.82-0.91) | Not reported | Distantly related |
| - Core - Ext | VAL  Maeda, 2019 | Multi-center observational cohort | Japan | GCS score ≤8 | Unfavorable outcome; 6mo | 511;  Unfav: 293 | Unfav:  Core:  0.81 (0.77–0.85)  Ext:  0.85 (0.80–0.89) | Not reported | Distantly related |
| - Core | VAL  Rached, 2019 | Multi-center observational cohort | Switzerland | ≥16 years;  Severe TBI (HAIS > 3) | Mortality; 14 days | 808;  Mortality: 240 | Mortality:  Core:  0.83 (0.80–0.86) | Not reported | Distantly related |
| - Core - Ext - Lab | VAL  Wongchareon, 2020 | Multi-center trial and observational cohort | South America | Severe TBI | Mortality, Unfavorable outcome; 6mo | 466;  Mortality: 177  Unfav: 264 | Mortality:  Core:  0.68 (0.63-0.73)  Ext:  0.73 (0.68-0.77)  Lab:  0.73 (0.68-0.78)  Unfav:  Core:  0.76 (0.71-0.80)  Ext:  0.78 (0.74-0.82)  Lab:  0.77 (0.73-0.81) | Hosmer-Lemeshow test: p-value  Mortality  Core  0.04  Intercept:0.08  Slope:0.98  Ext  0.05  Intercept:0.03  Slope:1.08  Lab  0.002  Intercept:0.05  Slope:1.05  Unfav  Core  0.00  Intercept:0.07  Slope:1.01  Ext  0.002  Intercept:-0.01  Slope:1.15  Lab  0.16  Intercept:0.11  Slope:1.04 | Distantly related |
| - Core | VAL  Camarano, 2021 | Multi-center observational cohort study | U.S., Canada; | GCS 3-12 | In-hospital mortality or discharge to hospice | 26.228;  Mortality: 10.919 | Mortality:  Core  0.863 (0.858-0.867) | Not reported | Distantly related |
| - Core | VAL  Camarano, 2021 | Multi-center observational cohort study | U.S., Canada; | GCS 3-14 | In-hospital mortality or discharge to hospice | Not reported | Mortality:  Core  0.865 (0.861-0.869) | Not reported | Distantly related |
| - Core | VAL  Elahi, 2021 | Single-center observational cohort study | Tanzania; | Mild, moderate and severe TBI; GCS 3-15 | Unfavorable outcome (GOS 1-3) | 2972;  Unfav: 327 | Unfav:  Core  0.821 (0.793-0.849) | Hosmer-Lemeshow test: p-value  <.001 | Distantly related |
| - Core - Ext - Lab | VAL  Dijkland,  2021 | Multi-center observational cohort study | Europe, Israel; ED, ward and ICU | Age ≥ 14;  GCS ≤ 12 | Mortality; 6mo  Unfavorable outcome; 6mo | Core  1173;  Mortality:  Unfav:  Ext  1030;  Mortality:  Unfav:  Lab  1006;  Mortality:  Unfav: | Mortality:  Core  0.81 (0.79-0.84)  Ext  0.85 (0.82-0.87)  Lab  0.85 (0.82-0.87)  Unfav:  Core  0.77 (0.74-0.80)  Ext  0.80 (0.78-0.83)  Lab  0.81 (0.78-0.84)  Mbc  Mortality:  Core  0.77 (0.75-0.80)  Ext  0.80 (0.76-0.82)  Lab  0.79 (0.77-0.83)  Unfav:  Core  0.78 (0.74-0.79)  Ext  0.80 (0.79-0.84)  Lab  0.81 (0.78-0.84) | Mortality  Core  Intercept:-0.74  Slope:1.20  Ext  Intercept:-0.73  Slope:1.23  Lab  Intercept:-0.37  Slope:1.32  Unfav  Core  Intercept:-0.10  Slope:0.97  Ext  Intercept:-0.03  Slope:1.01  Lab  Intercept:0.12  Slope:1.02 | Moderately related |
| **Yuan models** | | | | | | | | | |
| Yuan;   - A - B - C - D | DEV  Yuan, 2012 | Single-center cohort study | China | ≥ 18 years; GCS ≤ 12, for whom at least one computed tomography (CT) scan and laboratory examination was performed after injury. | Mortality, Favorable outcome;30 days and 6mo | 1016;  Mortality: not reported  Favorable: not reported | Mortality 30 days:  A: 0.71 (0.69-0.72)  B: 0.78 (0.74-0.81)  C: 0.87 (0.84-0.90)  D: 0.94 (not reported)  Fav 6mo:  A: 0.75 (0.73-0.76)  B: 0.80 (0.77-0.82)  C: 0.84 (0.81-0.86)  D: 0.92 (not reported) | Hosmer-Lemeshow test: p-value  A: 0.14  B: 0.89  C: 0.29  D: 0.87  A: 0.17  B: 0.72  C: 0.32  D: 0.41 | NA |
|  | VAL  Yuan, 2012 | Single-center cohort study | China | ≥ 18 years; GCS ≤ 12, for whom at least one computed tomography (CT) scan and laboratory examination was performed after injury. | Mortality, Favorable outcome; 30 days and 6mo | 203;  Mortality: not reported  Favorable: not reported | Mortality 30 days:  A: 0.84 (0.79-0.90)  B: 0.90 (0.86-0.94)  C: 0.90 (0.86-0.94)  Fav 6mo:  A: 0.87 (0.82-0.92)  B: 0.92 (0.88-0.96)  C: 0.89 (0.83-0.95) | Hosmer-Lemeshow test: p-value  Mortality 30days:  A: 0.43  Intercept: 0.35  Slope: 1.48  B: 0.22  Intercept: 0.65 Slope: 1.61  C: 0.05  Intercept:-0.99  Slope: 1.38  Fac 6mo:  A: 0.42  Intercept:-0.21  Slope: 1.54  B: 0.36  Intercept:-0.03  Slope: 1.74  C: 0.10  Intercept:-0.41  Slope:1.17 | Related |
| For the IMPACT prognostic models we calculated the mean AUC at development: Mortality Core 0.76, Extended 0.79, Lab 0.77 and Unfavorable outcome Core 0.77, Extended 0.80, Lab 0.79  *Calibration intercepts and slopes were calculated incorrectly. | | | | | | | | | |

| **Supplementary Table 8:** *Median AUC and IQR at development and validation for each model* | | | | | | | | | |
| --- | --- | --- | --- | --- | --- | --- | --- | --- | --- |
|  | **Overall** | | | **Mortality** | | | **Unfavorable outcome** | | |
| **Model** | **AUC dev** | **N val** | **AUC val** | **AUC dev** | **N val** | **AUC val** | **AUC dev** | **N val** | **AUC val** |
| APACHE II | 0.86 | 11 | 0.79 [0.74,0.80] | 0.86 | 10 | 0.79 [0.74,0.80] | NA | NA | NA |
| CRASH Basic | 0.83 | 26 | 0.83 [0.77,0.88] | 0.86 | 13 | 0.86 [0.80,87] | 0.81 | 13 | 0.80 [0.76,0.88] |
| CRASH CT | 0.83 | 30 | 0.84 [0.77,0.89] | 0.88 | 13 | 0.83 [0.79,0.89] | 0.83 | 17 | 0.84 [0.77,0.86] |
| Hukkelhoven | 0.78 | 14 | 0.83 [0.75,0.87] | 0.78 | 7 | 0.87 [0.78,0.88] | 0.80 | 6 | 0.78 [0.72,0.83] |
| IMPACT Core | 0.76 | 59 | 0.80 [0.76,0.82] | 0.76 | 30 | 0.80 [0.75,0.83] | 0.77 | 21 | 0.78 [0.76,0.81] |
| IMPACT Extended | 0.79 | 50 | 0.81 [0.78,0.85] | 0.79 | 22 | 0.82 [0.78,0.85] | 0.80 | 21 | 0.80 [0.78,0.84] |
| IMPACT Lab | 0.79 | 36 | 0.80 [0.77,0.84] | 0.77 | 16 | 0.79 [0.77,0.83] | 0.79 | 17 | 0.80 [0.76,0.84] |
| MPM0 | 0.82 | 2 | 0.47 [0.47,0.47] | 0.82 | 1 | 0.47 [0.47,0.47] | NA | NA | NA |
| MPM24 | 0.84 | 2 | 0.57 [0.57,0.57] | 0.84 | 1 | 0.57 [0.57,0.57] | NA | NA | NA |
| Nijmegen Clinical | 0.83 | 6 | 0.82 [0.81,0.82] | 0.84 | 2 | 0.84 [0.83,0.85] | 0.82 | 3 | 0.81 [0.71,0.81] |
| Nijmegen Comb | 0.89 | 4 | 0.83 [0.76,0.84] | 0.90 | 1 | 0.86 [0.86,0.86] | 0.80 | 2 | 0.76 [0.72,0.79] |
| Nijmegen CT | 0.83 | 4 | 0.78 [0.74,0.79] | 0.85 | 1 | 0.79 [0.79,0.79] | 0.76 | 2 | 0.74 [0.73,0.76] |
| Rotterdam CT | 0.75 | 6 | 0.77 [0.72,0.84] | 0.75 | 4 | 0.83 [0.76,0.85] | 0.75 | 1 | 0.72 [0.72,0.72] |
| SAPS II | 0.86 | 12 | 0.71 [0.62,0.73] | 0.86 | 11 | 0.71 [0.62,0.73] | NA | NA | NA |
| Signorini | 0.90 | 6 | 0.77 [0.68,0.84] | 0.90 | 3 | 0.84 [0.77,0.84] | 0.90 | 2 | 0.61 [0.61,0.61] |
| Yuan A | 0.73 | 3 | 0.85 [0.,85,0.86] | 0.71 | 1 | 0.84 [0.84,0.84] | 0.75 | 1 | 0.87 [0.87,0.87] |
| Yuan B | 0.79 | 3 | 0.91 [0.91,0.92] | 0.78 | 1 | 0.90 [0.90,0.90] | 0.80 | 1 | 0.92 [0.92,0.92] |
| Yuan C | 0.85 | 3 | 0.90 [0.89,0.90] | 0.87 | 1 | 0.90 [0.90,0.90] | 0.83 | 1 | 0.89 [0.89,0.89] |
